# A meta-analysis of vaccine efficacy from phase III clinical trials of approved vaccines against SARS-CoV-2 and variants

**DOI:** 10.1186/s12879-025-11289-4

**Published:** 2025-09-26

**Authors:** Dipesh Dhayfule, Yu-Heng Wu, Akram Ashyani, Ming-Chi Li, Chin-Shiang Tsai, Po-Lin Chen, Torbjörn E. M. Nordling

**Affiliations:** 1 Department of Mechanical Engineering, National Cheng Kung University, No. 1 University Rd., Tainan, 701 Taiwan; 2 Department of Internal Medicine, National Cheng Kung University Hospital, College of Medicine, National Cheng Kung University, No. 138 Sheng - Li Rd, Tainan, 701 Taiwan

**Keywords:** COVID-19, Meta-analysis, Phase III clinical trials, SARS-CoV-2, Vaccine efficacy

## Abstract

**Background:**

As we emerge from the COVID-19 pandemic and transition to a post-pandemic era, it is crucial to reflect on our experiences and prepare for future pandemics. Here we evaluate the impact of different methods for calculating the vaccine efficacy of COVID-19 vaccines, which has not been done previously.

**Methods:**

We conducted a meta-analysis of 38 approved COVID-19 vaccines using data from phase III clinical trials between May 4, 2020, and June 10, 2022. We analyze vaccine efficacy against multiple SARS-CoV-2 variants including the original strain, Alpha, Beta, Delta, and Kappa using multiple endpoints. Clinical endpoints are categorized into a tree structure including asymptomatic infection, symptomatic infection, mild to critical illness, and death. We employ re-estimated vaccine efficacies, including relative risk and Poisson regression with robust error variance, for equitable cross-vaccine comparisons.

**Results:**

We re-estimated 63 vaccine efficacies, revealing a 3% to 6% difference in five efficacies compared to the original study. Four efficacies exhibited lower bounds below the critical 50% threshold for the endpoint asymptomatic, symptomatic, moderate, and severe, contrary to the initial reports. However, efficacy consistently surpasses the 50% threshold against symptomatic COVID-19. Overall efficacies range from 34.2% to 100%, 50.3% to 100% against symptomatic, and 66.8% to 100% against severe, and 65% to 95% against variants.

**Conclusions:**

Our systematic classification of vaccine endpoints enables more statistically rigorous meta-analyses across studies. Beyond the quantitative results, our study emphasizes the need to standardize the estimation method for robust assessments of vaccine efficacy. We highlight the incompleteness of the knowledge about different vaccine efficacy in the middle of the pandemic, in particular the need to identify variants during the trials and report on multiple endpoints. We encourage all authors to publicly share their data, fostering additional impartial investigations. This data collection enables comparisons with real-world effectiveness data, enabling future studies of the predictive power of efficacy.

**Supplementary Information:**

The online version contains supplementary material available at 10.1186/s12879-025-11289-4.

## Introduction

A total of 38 vaccines were approved to combat SARS-CoV-2 and its variants, with the World Health Organization (WHO) granting emergency use listing (EUL) to 11 vaccines during the peak of the pandemic, before June 10, 2022. The purpose of this study is to provide a picture of the available knowledge at that time to identify shortcomings and discuss their potential impact on vaccine approval, in particular the method used to estimate vaccine efficacy and available data.

A US study with 3.4 million individuals found that BNT162b2 vaccine efficacy decreased from 88% (95% CI, 86-89%) to 47% (95% CI, 43-51%) during a period from December 14, 2020, to August 8, 2021 [1]. New variants, known for increased transmission and potential reinfection risks, led the WHO to classify them as Variants of Concern (VOC) and Variants of Interest (VOI) [2, 3]. The surge in Delta variant cases in July 2021 raised concerns globally, prompting considerations for booster doses [4]. Only three booster dose clinical trials were identified (AZD1222, BNT612b2, Soberana Plus) on June 10, 2022. The need for booster doses arises due to waning protection, reduced efficacy against VOCs, and incomplete protection in certain risk groups [5].

Zahid et al. [6] covered the clinical trial stages, vaccine progress, modes of action, and efficacy with data collected up to February 2021. Tregoning et al. [7] reviewed ten vaccines, four of which were WHO-approved and published in August 2021, emphasizing phase I-III trials efficacy and their current state, effectiveness, and modes of action. Fan et al. [8] focused on seven vaccines in phase III trials between January 24, 2020, and May 30, 2021, addressing safety and efficacy. Higdon et al. [9] assessed 15 vaccines in phase III and 8 for real-world effectiveness against SARS-CoV-2 infection and disease. Sobczak et al. [10] reviewed 17 randomized clinical trials of COVID-19 vaccines published before November 2, 2021 and focused on symptomatic infections, severe cases, hospitalizations and mortality rates by vaccine type. Yang et al. [11] examed efficacy against asymptomatic/symptomatic infections, severe disease, hospitalization and death across different variants and age groups between January 1, 2020 and September 12, 2022. However, these studies did not implement a systematic categorization of efficacy endpoints, nor did they provide a comparative analysis between originally reported efficacy values and re-estimated estimates. Recognizing this gap, there is a discernible need for a comprehensive review encompassing all approved vaccines alongside their respective phase III clinical trial outcomes.

In this meta-analysis, we discuss phase III clinical trials, post-phase III studies of approved vaccines, their booster dose vaccines, and methods to calculate vaccine efficacy and confidence interval. We classify endpoints according to severity by using the definitions provided in the clinical studies. We did our utmost to include all studies available as of June 10, 2022 and systematically assessed and reported the information using the same method to enable future study of the accuracy of this information. We also gather information on the clinical study’s financing source and the types of trial data that are publicly available for additional investigation. We cover how many countries had approved each vaccine, as well as the storage temperature and shelf life. The definitions of the measure endpoints from the various studies were reclassified according to WHO clinical progression scale. Finally, we calculate the vaccine efficacy of all vaccines in each measure endpoint category in order to demonstrate the overall effect of all vaccines in each category, as well as compare different methods for estimation of vaccine efficacy.

## Methods

**Fig. 1:**
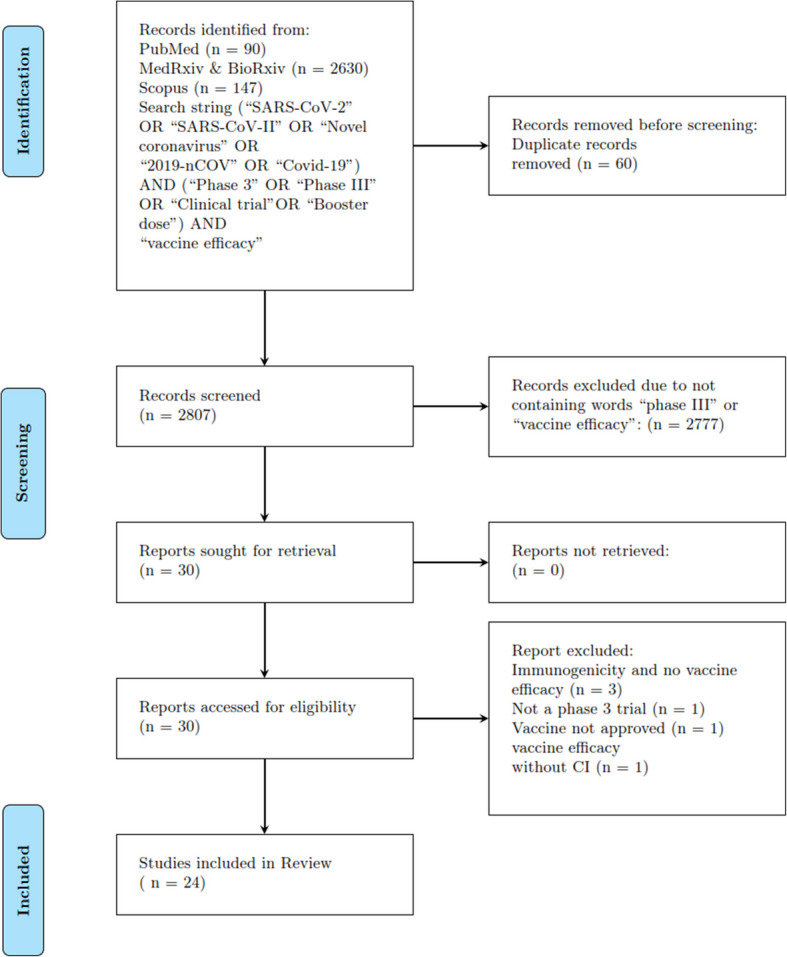
PRISMA flow diagram. The search string used in this study was (“SARS-CoV-2” OR “SARS-CoV-II” OR “Novel coronavirus” OR “2019-nCOV” OR “Covid-19”) AND (“Phase 3” OR “Phase III” OR “Clinical trial” OR “Booster dose”) AND “vaccine efficacy”

In adherence to the standards outlined in the Preferred Reporting Items for Systematic Reviews and Meta-Analyses (PRISMA) guidelines, a prompt review was undertaken [12]. We searched for vaccine efficacy phase 3 trials, booster dose, extension, and post-phase 3 trials vaccine efficacy peer-reviewed and pre-prints for all the approved vaccines. Our PRISMA flow diagram is shown in Fig. 1. We search through multiple databases: Medline, Web of Science, Embase, Scopus, and pre-print servers MedRxiv and BioRxiv. Our search string includes (“SARS-CoV-2” OR “SARS-CoV-II” OR “2019-nCOV” OR “Novel coronavirus” OR “Covid-19”) AND (“phase 3” OR “phase III” OR “Clinical trial” OR “booster dose”) AND “vaccine efficacy”. The search was conducted from 2020-05-04 to 2022-06-10, during the peak of the pandemic, resulting in the identification of 2970 articles. The title and abstract should meet the following criteria:

i) The focus of the study is on phase III clinical trial
ii) It is about vaccine efficacy against SARS-CoV-2 or its variants.

After all of the criteria are met, the studies are included in the full-text reading for the phase III clinical trial of the SARS-CoV-2 vaccine. After studying the titles, abstracts, and removing duplicates, we identified 30 articles. We excluded articles on the basis of the following criteria:

i) No report of vaccine efficacy against novel coronaviruses.
ii) Vaccine efficacy not reported after the second dose of a two-dose vaccine nor after the first dose of a one-dose vaccine.
iii) No confidence interval reported for the measured endpoints, unless it cannot be calculated.
iv) News articles and company published reports.
v) No vaccine approved by at-least one country.

After applying all the inclusion and exclusion criteria, we included 24 peer-reviewed articles in our meta analysis which include phase III trials, booster doses, and post-phase III vaccine efficacy studies. Our meta analysis also includes the approved vaccines, “Daughter vaccine”, which shares the same chemical formulation. For example, the Serum Institute of India’s “Covishield” vaccine is a daughter vaccine of the AZD1222 parent vaccine. Takeda-919 is the daughter vaccine of the mRNA-1273 parent vaccine. The covovax vaccine is the daughter vaccine of Novavax vaccine. The results of the parent vaccine’s phase III clinical trial were also applied to the daughter vaccine. That is for vaccines sharing the same formulation but manufactured under different trade names or jurisdictions, we treated efficacy estimates as interchangeable. While this assumption follows WHO guidelines on biological equivalence, slight differences in lot quality, cold-chain integrity, or population immunity may affect real-world outcomes. To show the overall effect of vaccine efficacy in each category, we added the number of participants and infected in the vaccine and placebo groups, respectively, to calculate the average vaccine efficacy and confidence interval of each measure endpoint. The endpoints of the studies conducted during the period when SARS-CoV-2 variants were dominating without determining the variant using genome sequencing of nose swab samples were marked as “mixed variants”.

### Vaccine efficacy calculation methods

**Fig. 2:**
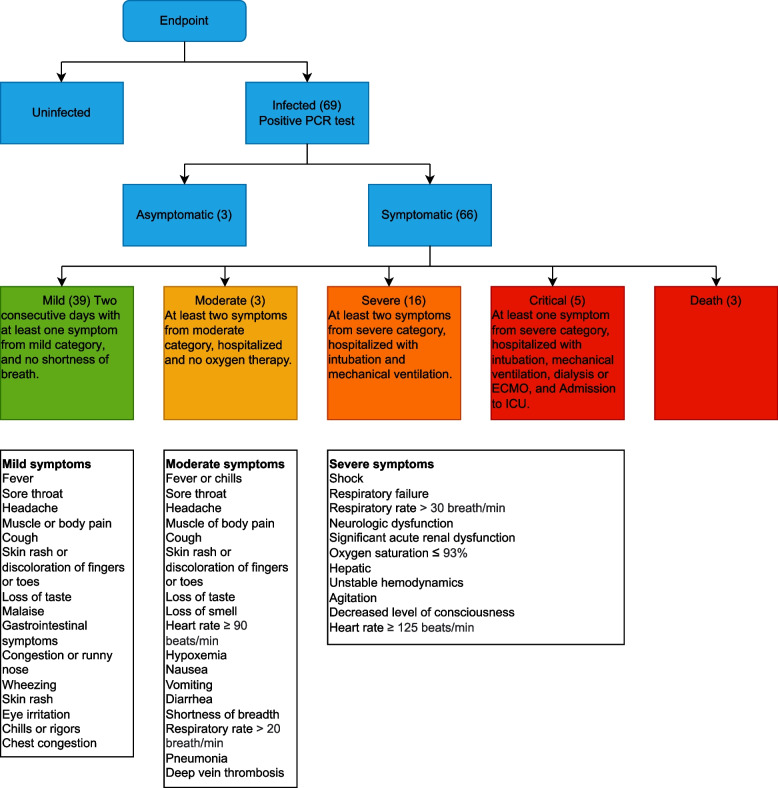
Classification of all the endpoints, including the criteria for each endpoint measure. Legends in green, yellow, orange, and red highlight the severity of the COVID-19 from low to high, respectively

In a clinical trial, the population is divided into two groups: the vaccination group and the placebo group. The vaccine group receives the number of doses of vaccine as per the instructions prescribed by the vaccine manufacturer, while the placebo group receives the same number of alum solution doses. We consider the observed number of vaccine $(x_{\text {ve}})$ and placebo $(x_{\text {pe}})$ events with total sum $(x_{\text {e}})$ in Table 1. In this case, the event is the selected endpoint of COVID-19 infection or the set including all more severe endpoints defined in Fig. 2. The non-event is the complementary set of endpoints, which in the later case is the set of all less severe endpoints. We denote the observed number of vaccine $(x_{\text {vn}})$ and placebo $(x_{\text {pn}})$ non-events with total $(x_{\text {e}})$, $x_{\text {v}}$ and $x_{\text {p}}$ are the sum of event and non-event individuals in vaccine and placebo groups respectively. Table 2 represents the parameter measures and methods used to calculate the vaccine efficacy in the respective studies. The vaccine efficacy (VE) in percentage is:

1 $$\begin{aligned} \text {VE} = (1 - R) \times 100\%. \end{aligned}$$

where *R* can either be relative risk, hazard ratio, odds ratio, incidence rate ratio or attack rate.

**Table 1:** Contingency table with observed counts of vaccine and placebo group

	Event	Non-event	Sum
Vaccine	$x_{\text {ve}}$	$x_{\text {vn}}$	$x_{\text {v}}$
Placebo	$x_{\text {pe}}$	$x_{\text {pn}}$	$x_{\text {p}}$
Sum	$x_{\text {e}}$	$x_{\text {n}}$	*x*

**Table 2:**
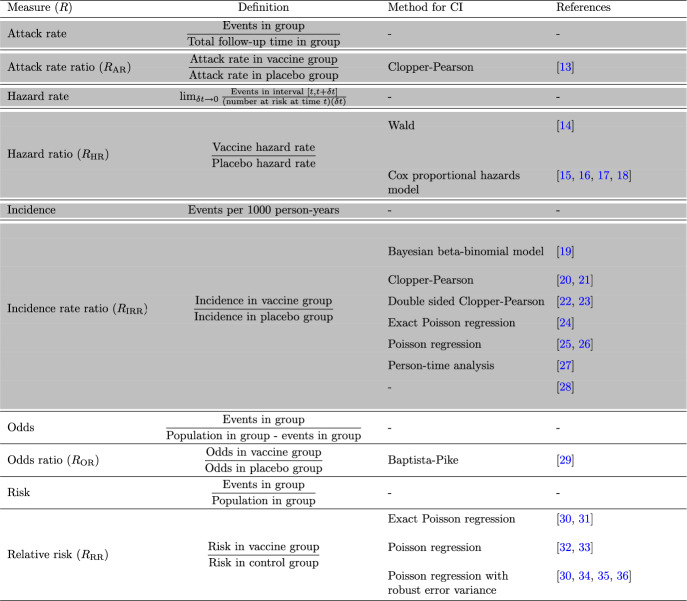
All methods with their definitions used to calculate vaccine efficacy and confidence intervals in clinical trial studies

The methods based on time-to-event data are highlighted with a shaded background

The risk ratio has a direct epidemiological interpretation as the proportionate change in average disease risk due to exposure. In contrast, the odds ratio lacks simple interpretation in terms of actual disease occurrence and can only be meaningfully interpreted when it serves as a risk ratio estimate [37]. For cohort studies where we can directly calculate meaningful prevalences or incidiences, we should use the risk ratio since it has a more intuitive interpretation, while odds ratios are more appropriate for case-control studies where true prevalences or incidences cannot be directly estimated [38]. The absence of time-to-event data in numerous studies led us to opt for the more straightforward implementation of Poisson regression with robust error variance for confidence interval estimation.

### Statistical analysis

Following the standard methodology outlined by [39], we estimate the relative risk as

2 $$\begin{aligned} \hat{R}_{\text {RR}} = \frac{x_{\text {ve}}/x_{\text {v}}}{x_{\text {pe}}/x_{\text {p}}} \end{aligned}$$

To estimate the vaccine efficacy and its confidence interval, we employed Poisson regression with robust error variance. The methodology is detailed in [40], which illustrates that the robust error variance effectively corrects for the overestimation problem in prospective studies with binary data. They introduced the variance of the relative risk estimated as

3 $$\begin{aligned} \widehat{Var}\left[ \ln \hat{R}_{\text {RR}}\right] = \frac{1}{x_{\text {ve}}}- \frac{1}{x_{\text {v}}}+\frac{1}{x_{\text {pe}}}-\frac{1}{x_{\text {p}}}. \end{aligned}$$

When one of the denominators is zero, the calculation of confidence intervals becomes problematic. A common solution to address zero counts in exposure groups when calculating confidence intervals is to add 0.5 to $x_{\text {ve}}$, $x_{\text {pe}}$, $x_{\text {vn}}$, and $x_{\text {pn}}$ [41]. However, this method may result in a re-estimated VE that deviates substantially from the reported value and may produce overly conservative lower bounds for the confidence interval. Therefore, in the main text, we exclude these cases without using the 0.5 addition method, while presenting the alternative results with the 0.5 addition method in the Supplementary Materials. All the statistical analyses, including the relative risk calculations and confidence interval estimations, were implemented using Python.

The meta-analysis of both the random effects model (DerSimonian-Laird) and the fixed effects model (Mantel-Haenszel) was performed by using the open-source Python package PythonMeta [42]. Heterogeneity was assessed using $I^2$, Chi-square (Cochran’s *Q*-test), and between-study variance $\tau ^2$ (DerSimonian and Laird method). Publication bias, seen as asymmetry in the funnel plots, was evaluated using Egger’s test.

### Data collection

We used data from McGill University’s COVID-19 vaccine tracker (COVID-19 Vaccine Tracker) to compile a list of approved vaccines in each country. We also collected information such as the number of countries where the vaccine has been approved for use, the number of clinical trials that have been conducted, the platform used, clinical trial funding source, type of data publicly available, and the amount of doses required. We collected the following information from the identified studies: author, study design, vaccine name, number of participants in vaccine and placebo groups, number of infected in vaccine and placebo groups, statistical methods, vaccine efficacy, measures, countries where the trial was conducted, storage temperature, shelf-life, follow-up duration, funding organization and data available. Endpoints of vaccine efficacy against documented infection, symptomatic, asymptomatic, mild, moderate, severe, and death were all included for SARS-CoV-2 and its variants. We searched the vaccine manufacturing company webpage, media articles, and other sources to find interim vaccine efficacy for vaccines whose vaccine efficacy has not yet been published in phase III clinical trials. We counted the number of studies of vaccine efficacy against the SARS-CoV-2 and its variants using the following criteria:

i) The vaccine efficacy reported in an article against the SARS-CoV-2 or its variants.
ii) If an article lists VE for several vaccines, each one is counted separately.
iii) If a single vaccine has multiple VE against multiple variants, each one is counted separately.

The study methods were read and evaluated in order to determine the number of participants in each cohort, age, gender, and protocols. The Statistical analysis section was read and analysed in order to identify the method for calculating vaccine efficacy, its confidence interval (CI), and the definition of measures. We conducted a Google search with the name of the vaccine checked, vaccine manufacturing company website, Wikipedia pages, and media reports to identify the interim vaccine efficacy of the approved vaccines that have yet to release their phase III clinical trial studies. This search was conducted through June 10, 2022.

## Results

Our data for each vaccine contains the details of the manufacturing company or organization, platform, storage temperature, shelf life, number of clinical trials conducted in different countries, and the number of countries that have approved it for usage. Table 3 describes thirty-eight vaccines approved by at least one of the countries for use. The number of clinical trials is the sum of Phase I, Phase II, and Phase III trials of the respective vaccines. Our meta-analysis focuses only on phase III clinical trials and post-phase III clinical studies of approved vaccines and their booster doses.

**Table 3:** Vaccine details (Updated to 2022-04-15)

Vaccine name	Company name	Platform	Doses	Storage (°C)	Shelf life (months)	Clinical trials/Countries	Countries approved	References
BNT162b2${*\dagger \text {T}}$	Pfizer-BioNtech	RNA	2	−80 - −60	6	80/26	146	[19]
AZD1222$^{*}$	Oxford Univ.\AstraZeneca	NRVV	2	2-8	6	63/33	140	[35, 43, 44]
Ad26.COV2S$^*\dagger$	Johnsson & Johnsson	NRVV	1	−25 - −15	24	23/23	111	[31]
Covilo${^*}$	SinoPharm (Beijing)	Inactivated	2	2-8	24	29/12	91	[20]
mRNA-1273$^{*\dagger }$	Moderna	RNA	2	−20	4	63/22	86	[16]
Sputnik V$^{\dagger }$	Gamaleya SRI	NRVV	2	−18	6	24/7	74	[29, 43]
CoronaVac${^*}$	SinoVac	Inactivated	2	2-8	12	37/9	56	[14]
Covisheild$^{*\text {D}}$	Serum Inst. of India	NRVV	2	2-8	6	4/1	49	[45]
NVX-CoV2373$^{*\dagger }$	NovaVax	Recombinant protein subunit	2	2-8	9	16/12	37	[34]
Sputnik light$^{\dagger }$	Russian health ministry	NRVV	1	−18	6	7/3	26	[46, 47]
BBV152 (Covaxin)${^*}$	Bharat biotech	Inactivated	2	2-8	6	14/2	14	[13, 43]
Ad5-nCoV (Convidecia)${^*}$	Cansino biologist	NRVV	1	2-8	12	13/6	10	[17]
CIGB-66 (Abdala)	Center for Genetic engineering and biotechnology in Cuba	Protein subunit	3	2-8	-	5/1	6	[47]
Zifivax	Anhui Zhifei Longcom	Protein subunit	2	2-8	-	20/5	4	[25]
EpiVacCorona	FBRI	Protein subunit	2	2-8	-	4/1	4	[47]
Soberana 02$^{\dagger }$	Finlay Institute	Protein subunit	2	2-8	6	4/2	4	[18]
COVOVAX$^{*\text {D}\dagger }$	SII	Protein subunit	2	2-8	6	2/1	5	[47]
MVC-COV1901	Medigen	Protein subunit	2	2-8	-	14/4	3	[47]
VLA2001	Valneva	Inactivated	2	2-8	6	6/3	3	[47]
KoviVac	Chumakov Center	Inactivated	2	2-8	-	3/1	3	[47]
Inactivated (Vero cells)	Sinopharm (Wuhan)	Inactivated	2	2-8	12	9/7	2	[47]
Corbevax$^\text {T}$	Biological E limited	Protein subunit	2	2-8	-	7/1	2	[47]
KCONVAC	Minhai biotechnology	Inactivated	2	2-8	-	5/1	2	[47]
QazCovid-in	Kazakhstan RIBSI	Inactivated	2	2-8	6	3/1	2	[47]
Recombiant SARS-CoV-2	National vaccine & SII	Protein subunit	2	-	-	3/2	1	[47]
Turkovac	Health institute of Turkey	Inactivated	2	-	-	8/1	1	[47]
Covax-19 (SpikoGen)	Vaxine/ChinaGen Co.	Protein subunit	2	-	-	4/1	1	[47]
Covifenz	Medicago	VLP	2	-	-	6/6	1	[47]
COVIran Barekat	Shifa PIC	Inactivated	2	2-8	-	6/1	1	[47]
Razi Cov Pars	Razi vaccine & SII	Protein subunit	3	-	-	5/1	1	[47]
Soberana Plus${\dagger }$	Finlay Institute	Protein subunit	1	2-8	4	5/1	1	[18]
ZyCov-D$^\text {T}$	Zydus Cadila	DNA	3	30	3	5/1	1	[28]
Fakhravac	ODIR IRAN	Inactivated	2	-	-	3/1	1	[47]
Aurora-CoV	Vector state research	RNA	2	-	-	3/1	1	[47]
TAK-919$^\text {D}$	Takeda	RNA	2	−20	4	2/1	1	[47]
Noora	Bagheiat-allah University of Medical Sciences	Protein subunit	2	-	-	3/1	1	[47]
TAK-019$^\text {D}$	Takeda	Protein subunit	2	-	-	2/1	1	[47]

The vaccines are listed in descending order of the number of countries that have approved them. The vaccines marked with $*$ are approved by WHO and those marked with $\dagger$ have released booster doses or is a booster vaccine. The vaccines labelled with “D” share the same chemical formulation as previously approved vaccines. Vaccines labelled with the letter “T” have been approved for use in children aged 12 and up. Abbreviation SII — Serum Institute of India, NRVV — Non Replicating Viral Vector

### Comparison of vaccine efficacy methods

We classify the endpoints by taking the definitions of endpoint measures from all the studies and reclassifying them using the WHO clinical progression scale. The measure adjusted vaccine efficacy endpoint mild contains endpoints from studies such as “infection”, “documented infection”, “confirmed covid-19”, “symptomatic”, “symptomatic infection”, “symptomatic covid”, “covid-19 illness”, and “symptomatic disease”. The adjusted measure endpoint moderate contains only “symptomatic-moderate” from the clinical trials studies. Measure endpoints from clinical studies “severe”, “symptomatic-severe”, “severe covid-19”, “incident severe”, “moderate-severe”, and “severe disease” were reclassified as adjusted vaccine efficacy endpoint severe. We classified the measure endpoint of vaccine efficacy “symptomatic-severe-critical”, “ICU” and “severe or critical” into adjusted vaccine efficacy endpoint measure critical. Figure 2 represents the 69 endpoints classification of vaccine efficacy against SARS-CoV and its variant in clinical trials. The figure also contains a detailed description of the endpoint measures defined in the studies. The severity of the endpoints is represented by the colour of the leaves in the classification tree, with green being the least severe and red being the most severe. 66 of the 69 infected endpoints were symptomatic infection, while the remaining 3 were asymptomatic infection. We further categorize these 66 symptomatic infections based on their severity as mild (39), moderate (3), severe (16), critical (5) and death (3) endpoints respectively.

**Fig. 3:**
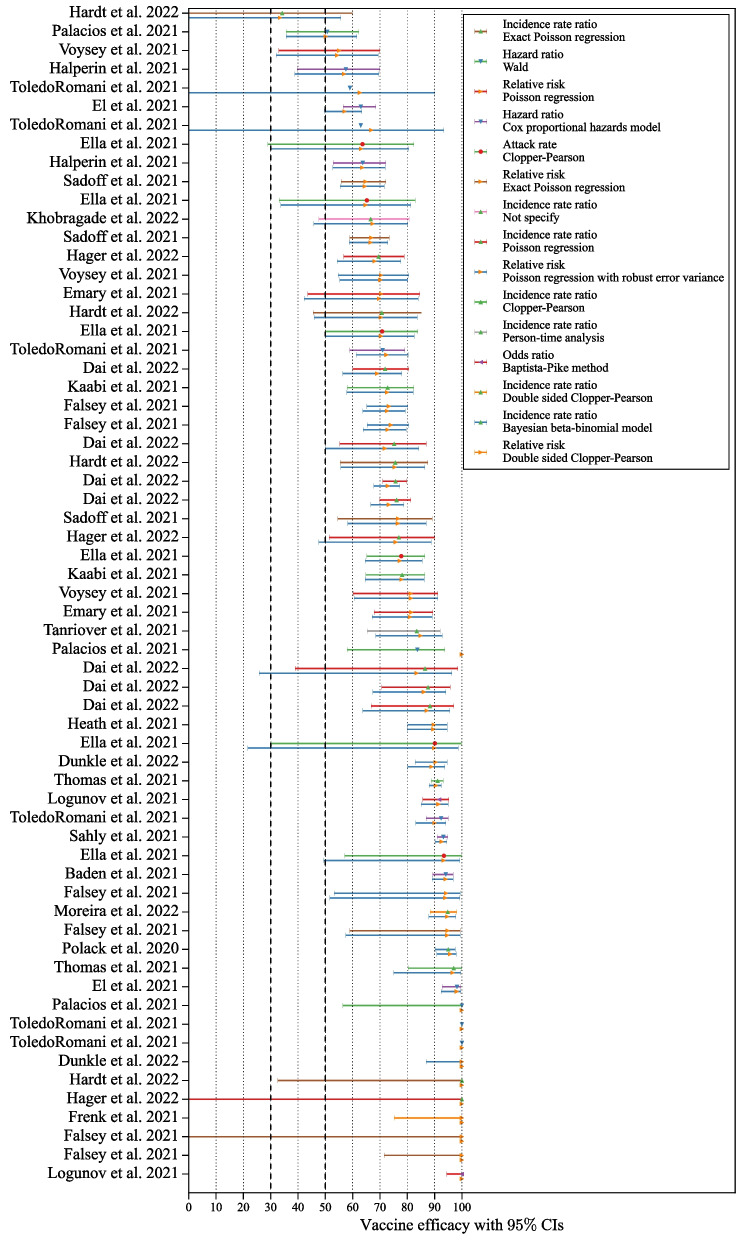
Vaccine efficacy and its confidence interval from the studies (the first line in each study) compared against the reproduced results (the second line in each study). The vaccine efficacies and its confidence intervals are reproduced using relative risk and Poisson regression with robust error variance

The re-estimation of 63 vaccine efficacies and associated confidence intervals was conducted using relative risk and Poisson regression with robust error methods. Figure 3 visually compares these re-estimated values across different vaccines. Two vaccine efficacies for vaccine name and endpoint reported by Toledo-Romaní et al. [18] do not include the confidence interval, while our re-estimation estimates a wide range of uncertainty, spanning from around 0% to 90%. The WHO international randomised trial of candidate vaccines against COVID-19 [48] specifies that a vaccine to be successful needs the vaccine efficacy point estimate to be at least 50% in placebo-controlled efficacy trials, with a sequential-monitoring-adjusted 95% lower bound confidence interval of at least 30%. We use these same criteria as critical threshold to evaluate vaccines. For 4 vaccines, our results demonstrate that their 95% lower bounds are below the 50% whereas the initial study reported the lower bounds of their efficacies as higher than the critical threshold of 50%. Out of the 11 efficacies reported as 100%, 3 initially lacked confidence intervals, and 7 of them have a wide spread of confidence intervals, with the lower bounds falling below 50%.

The differences between the original vaccine efficacy and its lower bound, and our re-estimated values are compared and displayed in Figs. 4 and 5. If the original study did not provide the confidence interval or the number of infected individuals in the vaccine group is zero then the data was excluded from the confidence interval analysis. Additional comparison results can be found in Supplementary Figures 1 to 11.

**Fig. 4:**
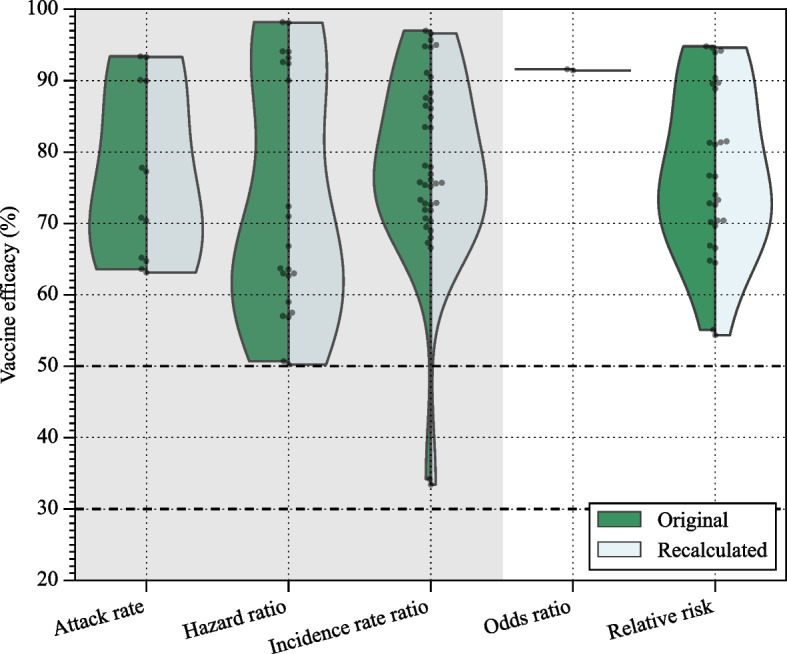
Violin plot illustrating the comparison of the original vaccine efficacy vs our re-estimation using the relative risk method. The mean is marked with a triangle, and each dot represents one data point. The dashed lines in the violin plot indicate the first, second, and third quartiles. The original methods utilizing time-to-event data are indicated by the shaded area. The critical thresholds of 50% and 30% are indicated by the dashed lines

**Fig. 5:**
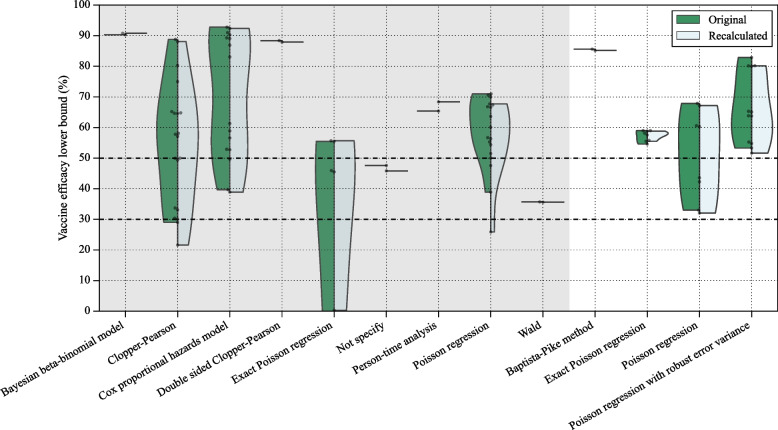
Violin plot illustrating the comparison of the original vaccine efficacy lower bound vs our re-estimation using the Poisson regression with robust error method. The mean is marked with a triangle, and each dot represents one data point. The dashed lines in the violin plot indicate the first, second, and third quartiles. The original methods utilizing time-to-event data are indicated by the shaded area. The critical thresholds of 50% and 30% are indicated by the dashed lines

### Efficacy of vaccines in clinical trials

**Fig. 6:**
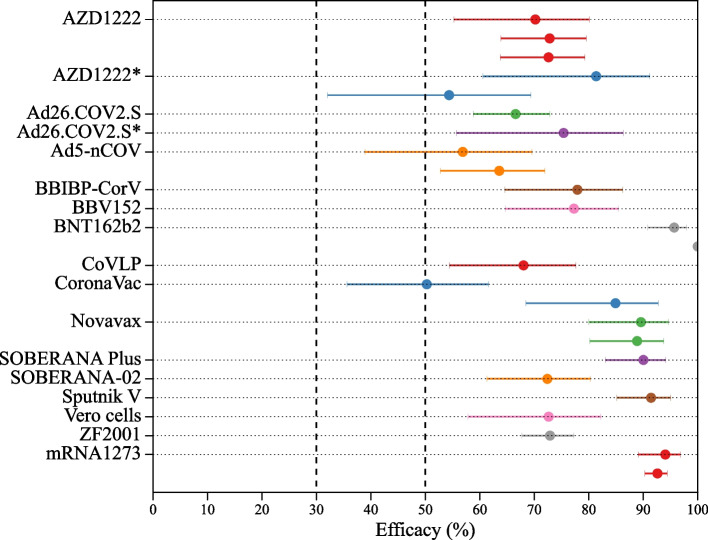
Vaccine efficacy against SARS-CoV-2 induced symptomatic endpoint with 95% confidence interval. The vaccines marked with * are the booster-dose vaccine

Figure 6 represents the vaccine efficacy of 15 vaccines and their booster dose with 25 endpoints against symptomatic infection/disease. In the phase 3 clinical trial, BNT162b2 demonstrated maximum efficacies of 100% and of 95.7% (95% CI, 90.8-98.0%). The BNT162b2 vaccine trial had the most participants (21720 in the vaccine and 21728 in the placebo groups) of any phase III clinical trial [19]. The mRNA-1273 vaccine showed a vaccine efficacy of 94.1% with 95% CI, 89.1-96.8%. BNT162b is the only vaccine that has completed a clinical phase trial with participants ranging in age from 12 to 15 years old. The trial enrolled 1131 participants in the vaccine group and 1129 participants in the placebo group. The vaccine demonstrated overall vaccine efficacy of 100% with no events occurring in the vaccine group versus 16 events in the placebo group [22]. The overall number of participants in the vaccine group across all trials that we report on is 295051 versus 230712 in the placebo group. The total number of participants infected in the vaccine group is 1009, whereas it in the placebo group is 4046 across all studies including all vaccines. The average vaccine efficacy of 16 vaccines against symptomatic SARS-CoV-2 is 80.49% (95% CI 79.1-81.79%).

**Fig. 7:**
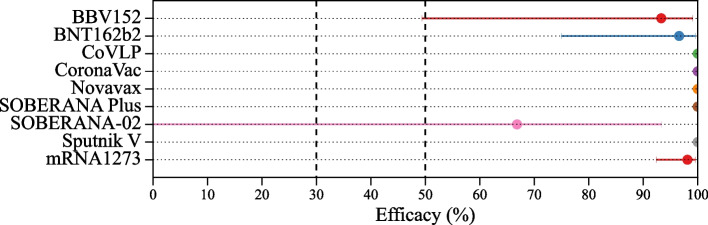
Vaccine efficacy against SARS-CoV-2 induced severe endpoint with 95% confidence interval

Figure 7 presents the vaccine efficacy of 12 different vaccine against severe SARS-CoV-2 obtained from the phase III clinical trials. Five vaccines reported efficacy of 100%, Sptutnik V [29], Coronavac [14], mRNA-1273 [16], BBIBP-CorV [20], Vero cells [20] and booster dose vaccine Soberana Plus [18]. The average vaccine efficacy against all severe endpoints is 97.66% (95% CI, 94.75-98.96%) with 6 events in the vaccine group versus 230 events in the placebo group.

**Fig. 8:**
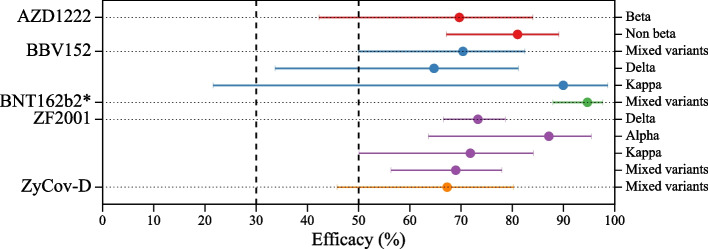
Vaccines efficacy against variants of SARS-CoV-2 induced symptomatic endpoint with 95% confidence interval. The vaccine marked with * is a booster dose vaccine

Figure 8 presents vaccine efficacy reported for SARS-CoV-2 variants spread during the phase III clinical trials. We obtained 11 endpoints for four vaccines and a booster dose. The BBV152 vaccine had the highest vaccine efficacy of 90.0% with 95% CI, 21.6-99.7% against the Kappa VOI, followed by ZF20001, which had a vaccine efficacy of 87.1% with 95% CI 63.6-95.5% against the delta variant [13, 34]. The booster dose of BNT162b2 vaccine showed vaccine efficacy of 94.7% with 95% CI, 88.0-95.5%. The average vaccine efficacy of BBV152, Novavax, and ZF2001 against the delta variant is 72.43% with 95% CI 65.92-77.7%. The average vaccine efficacy against the mixed variant is 76.66% with 95% CI 70.53-81.52% against the symptomatic endpoint.

Detailed information regarding vaccine efficacy against other categories, including asymptomatic, moderate, documented infection, hospitalization, ED, ICU, critical, and death, is presented in the supplementary materials.

### Meta-analysis

Statistical tests for asymmetry typically require 10 or more studies to achieve adequate power [49]. Therefore, we conducted meta-analyses only for vaccine efficacy endpoints with a sufficient number of studies. The symptomatic endpoint included 25 studies, while the severe endpoint had 9 studies, approaching but not reaching the threshold of 10. To expand our analysis, we introduced a ‘severe+’ endpoint that combines severe, critical, and death endpoints, yielding 18 studies. The detailed numbers of data points for each endpoint are presented in Supplementary Tables 1 through 10.

Tables 4 and 5 summarize the meta-analysis results for both the symptomatic and severe+ endpoints. We compared both fixed-effect and random-effect models, analyzing the weights and summary statistics including $\tau ^2$, $I^2$, Q, P (heterogeneity test), and Z (overall effect test). Higher values of $\tau ^2$, $I^2$, and *Q* indicate greater heterogeneity in the data.

**Table 4:** Summary of meta-analysis of vaccine efficacy for SARS-CoV-2 symptomatic endpoint, including Risk Ratio (RR) with 95% Confidence Intervals, weights of fixed effects (FE), random effects (RE), and meta-analysis summary statistics

Vaccine name [ref]	RR (95%CI)	FE (%)	RE (%)
AZD1222 [35]	0.30 (0.20–0.45)	2.40	4.28
AZD1222 [30]	0.27 (0.20–0.36)	4.17	4.56
AZD1222 [30]	0.27 (0.21–0.36)	4.32	4.57
AZD1222* [35]	0.46 (0.31–0.68)	1.82	4.30
AZD1222* [35]	0.19 (0.09–0.39)	1.05	3.31
Ad26.COV2.S [31]	0.33 (0.27–0.41)	8.34	4.70
Ad26.COV2.S* [24]	0.25 (0.14–0.44)	1.31	3.77
Ad5-nCOV [17]	0.43 (0.30–0.61)	2.49	4.42
Ad5-nCOV [17]	0.36 (0.28–0.47)	5.03	4.61
BBIBP-CorV [20]	0.22 (0.14–0.35)	2.26	4.10
BBV152 [13]	0.23 (0.15–0.35)	2.52	4.18
BNT162b2 [19]	0.04 (0.02–0.09)	3.86	3.28
BNT162b2 [22]	0.03 (0.00–0.50)	0.39	0.63
CoVLP [26]	0.32 (0.22–0.46)	2.98	4.41
CoronaVac [27]	0.15 (0.07–0.32)	0.99	3.33
CoronaVac [14]	0.50 (0.38–0.64)	4.03	4.62
Novavax [34]	0.10 (0.05–0.20)	2.29	3.59
Novavax [36]	0.11 (0.06–0.20)	2.00	3.80
SOBERANAPlus [18]	0.10 (0.06–0.17)	3.63	3.94
SOBERANA-02 [18]	0.28 (0.20–0.39)	3.70	4.45
SputnikV [29]	0.09 (0.05–0.15)	2.22	3.88
Verocells [20]	0.27 (0.18–0.42)	2.26	4.21
ZF2001 [25]	0.27 (0.23–0.32)	13.84	4.76
mRNA1273 [15]	0.07 (0.06–0.10)	17.71	4.59
mRNA1273 [16]	0.06 (0.03–0.11)	4.40	3.71
Total		Fix	Rand
Fixed Effects	0.22 (0.21–0.24)		
Random Effects	0.20 (0.16–0.26)		
Heterogeneity measures:			
$$\tau ^2=0.306$$			
$$I^2=90.07\%$$			
Q(Chi-square)=241.67 (p $\le$ 0.001)			
Overall effect test: z=42.36 (Fixed), z=13.02 (Random), p $\le$ 0.001			
Egger’s test: t=−3.26, p=0.022

**Table 5:** Summary of meta-analysis of vaccine efficacy for SARS-CoV-2 severe+ which includes severe, critical, and death endpoints, including Risk Ratio (RR) with 95% Confidence Intervals, Fixed Effects (FE), Random Effects (RE), and meta-analysis summary statistics

Vaccine name [ref]	RR (95%CI)	FE (%)	RE (%)
AZD1222 [30]	0.03 (0.00–0.49)	3.05	3.02
AZD1222 [30]	0.05 (0.01–0.42)	3.23	4.98
AZD1222 [30]	0.16 (0.01–3.96)	0.54	2.48
Ad26.COV2.S [31]	0.23 (0.13–0.42)	15.94	14.71
Ad26.COV2.S* [24]	0.05 (0.00–0.95)	2.34	3.02
BBV152 [13]	0.07 (0.01–0.51)	3.98	5.13
BNT162b2 [21]	0.03 (0.00–0.25)	7.89	5.24
CoVLP [26]	0.14 (0.01–2.76)	0.93	2.83
CoronaVac [14]	0.08 (0.00–1.34)	1.74	2.97
Novavax [36]	0.06 (0.00–1.03)	1.60	2.90
SOBERANAPlus [18]	0.08 (0.00–1.41)	1.70	2.97
SOBERANAPlus [18]	0.06 (0.00–1.05)	2.23	3.02
SOBERANA-02 [18]	0.37 (0.10–1.41)	2.13	8.62
SOBERANA-02 [18]	0.33 (0.07–1.64)	1.60	6.99
SputnikV [29]	0.01 (0.00–0.13)	8.22	3.10
ZF2001 [25]	0.17 (0.04–0.74)	3.20	7.56
ZF2001 [25]	0.14 (0.06–0.33)	11.47	12.32
mRNA1273 [15]	0.02 (0.00–0.08)	28.21	8.15
Total		Fix	Rand
Fixed Effects	0.09 (0.07–0.13)		
Random Effects	0.10 (0.06–0.17)		
Heterogeneity measures:			
$$\tau ^2=0.435$$			
$$I^2=39.24\%$$			
Q(Chi-square)=27.98 (p = 0.045)			
Overall effect test: z=13.88 (Fixed), z=8.31 (Random), p $\le$ 0.001			
Egger’s test: t=−2.76, p=0.014			

## Discussion

Each study used various methods to determine vaccine efficacy and their confidence intervals. To compare different vaccines, we re-estimated their efficacy and confidence intervals using the simplest relative risk and Poisson regression with a robust error method on the collected data (Fig. 3). The efficacy estimates in each study were adjusted in different ways. The largest discrepancies between the original and recalibrated estimates for the relative risk method occurred when the original studies adjusted relative risk based on age groups, follow-up durations, and risk periods [30–33, 35, 36]. As a result, our estimates differ from those in the original studies, even when they used the same methods for calculating efficacy and confidence intervals.

Our approach may create a biased estimate of the vaccine efficacy (VE) because the risk ratio method assumes that all participants have equal follow-up time. In other words, the risk ratio method assumes a fixed (or similar) follow-up window for each participant. This assumption may not hold true in all cases, as participants may have varying follow-up periods. A more accurate approach would be to use time-to-event analysis, which takes into account the different follow-up times for each participant. However, the majority of the studies do not report the necessary information for time-to-event analysis, including the studies where the relative risk was recalibrated, which forces us to use the more straightforward method. Despite this limitation, this method still provide valuable insights into vaccine efficacy, albeit with the potential for bias in the estimates.

While vaccine efficacy generally remained close to the original research estimates, we consistently observed lower vaccine efficacy when using relative risk. The application of Poisson regression with robust error variance resulted in reproduced confidence intervals closely mirroring the original study but with a shift due to changes in vaccine efficacy. Notably, when the vaccine group lacked diseased individuals, the Poisson regression with robust error variance failed to compute the confidence interval, indicating 100% vaccine efficacy without an error bar. A commonly used solution in such cases is the addition of $0.5$ to all cells of the contingency table to enable computation of standard errors and finite bounds [41]. While this continuity correction resolves computational issues, it introduces bias, often lowering the point estimate and inflating the confidence interval–especially in low-event settings. Alternative results using the $0.5$ continuity correction are reported in the Supplementary Materials for transparency and completeness. Some reproduced vaccine efficacy and confidence intervals significantly deviated from the original study, suggesting a notable bias in comparing estimations across different studies. Notably, the Hazard ratio and Incidence rate ratio exhibit a wider range of difference compared to the relative risk method. When recalculating the vaccine efficacy of a study that utilized the Hazard ratio [14], we observed the maximum difference of $16.3\%$. This discrepancy arose because the event count in the vaccine group was zero. Given that our method for recalculating vaccine efficacies, along with their confidence intervals, encounters problems with zero events–as previously discussed–we excluded this data point in the following discussion. By excluding the zero confidence interval data points, we observed five datapoints that revealed 3% to 6% differences, which are from ZF2001 (3.1%, 3.4%) [25], SOBERANA-02 (3.8%, 3.7%) [18], and mRNA1273 (6.0%) [15].

Two of them used the incidence rate ratio, i.e. time-to-event data, and the other three used the hazard ratio. A similar pattern can be found in Fig. 5. The bigger differences between the original study and our estimation happened in the methods using time-to-event data, i.e. the shaded area. Furthermore, our results reveal that the lower bounds of efficacies for four vaccines are below the 50% threshold in contrast to the initial study which reported their lower bounds as higher than 50%. Consequently, we assert the importance of employing uniform methods for determining vaccine efficacies and confidence intervals to facilitate meaningful comparisons of study results.

We also re-estimated the vaccine efficacy against different variants, as shown in Fig. 8. While all vaccine efficacies against variants exceed 50%, among the 11 endpoints, 5 have 95% CI lower bounds below 50% (AZD1222 against Beta, BBV152 against mixed variants, and Delta, and ZyCov-D against mixed variants), and 1 falls below 30% (BBV152 against Kappa).

Our meta-analysis results (Tables 4 and 5) show heterogeneity in the symptomatic endpoint but not in the severe+ endpoint, with the Z test indicating significant overall effects. The random-effects model revealed that the effect size for the symptomatic endpoint (0.2) was twice that of the severe+ endpoint (0.1). The fixed-effects model yielded similar results to the random-effects model. A 0.02 difference was found in the symptomatic endpoint (95% CI difference, lower bound 0.05, upper bound 0.02) and a 0.01 difference in the severe+ endpoint (95% CI difference, lower bound 0.01, upper bound 0.04). Both endpoints showed Egger’s test p-values below 0.05, suggesting the presence of publication bias, with corresponding funnel plots available in Supplementary Section 4.

While reviewing all published studies, we also looked for the funding source of the clinical trials, as well as what kind of clinical trial data is available and how to access it. We discovered that the vaccine-developing organization/companies/research & development institute financed more than half of the published clinical studies. Approximately 90% of clinical trial publishers have embraced the practice of making clinical trial data accessible, which can be obtained after securing permission from the corresponding author, either via email or by submitting a request through the website. Among these publishers, 13 facilitate data access through email requests, 8 provide data access through a designated URL, and 6 do not offer accessible data.

We highlight the incompleteness of the knowledge about different vaccines efficacy in the middle of the pandemic, in particular the need to identify variants during the trials and report on multiple endpoints. Our work to recalculate the vaccine efficacy would have been easier if each author would have shared a table with observed counts in the vaccine and placebo groups and also the time-to-event data.

Our study has several limitations that should be noted. Access to data from the articles was limited, as we focused on publicly available phase III clinical trials. This research covered studies conducted during the peak of the pandemic between May 4, 2020, and June 10, 2022, resulting in a specific cutoff date. The absence of enrollment and exclusion data posed challenges for our risk of bias analysis. Furthermore, the limited sample size affected the statistical power for endpoints such as critical illness and death. To address this limitation, we combined different endpoints into the severe+ endpoint in the meta-analysis. This approach highlights the advantages of our endpoint classification system shown in Fig. 2. For the meta-analysis, we utilized the open-source Python package PythonMeta [42] that currently lacks certain sensitivity analysis capabilities, such as the trim-fill method.

## Conclusion

We conducted a comprehensive meta-analysis of SARS-CoV-2 vaccine efficacy, concentrating on the thirty-eight approved vaccines in phase III clinical trials, post-phase III, and extended studies published before June 10, 2022. Our approach involved recalculating vaccine efficacy and confidence intervals using the provided data, including the number of individuals in the vaccine and placebo groups and the incidence of COVID-19 in each group. To our knowledge, none of the existing vaccine efficacy studies have systematically compared efficacy against SARS-CoV-2 using the same estimation method, emphasizing the unique contribution of our analysis in this regard. We re-estimated 63 vaccine efficacies by combining the relative risk and Poisson regression with robust error variance, identifying six vaccines with differences exceeding 3% between our estimations and the original study, with the maximum difference reaching 6%. The re-estimation unveiled lower bounds of efficacies for four vaccines, dipping below the 50% threshold, contrary to the initial study reports. The average vaccine efficacy of all vaccines is 80.5% (95% CI, 79.1-81.8%) against symptomatic infection. The average vaccine efficacy is 97.7% (95% CI, 94.7-98.9%) against severe and 86% (95% CI, 77.6-91.3%) against the critical endpoint. We found that 42% of the reported vaccine endpoints had a greater than 90% vaccine efficacy. Twelve vaccines including booster dose vaccines have demonstrated 100% vaccine efficacy against SARS-CoV-2 and beta variant in phase III clinical trials.

Our meta-analysis demonstrated the importance of a standardized endpoint classification system. The classification into symptomatic and severe+ endpoints enabled robust statistical analyses with 25 and 18 studies, respectively. This approach proved particularly valuable when individual endpoints such as critical illness or death lacked sufficient statistical power due to limited sample sizes. Our findings underscore that implementing such a classification system early in a pandemic can facilitate more reliable vaccine efficacy assessments.

Doing this analysis, composing, and publishing this report took so long that it had no impact on the decision-making during the pandemic. However, by preparing a pipeline and guidelines on what information to share with standard formats, it could be speeded up in future pandemics. Thus this highlights the need for preparation. This report also gives a picture of the incompleteness of knowledge even after systematic analysis that existed in the midst of the pandemic when key decisions on vaccination had to be taken. This report also enables future studies comparing the predictive power of vaccine efficacy on real vaccine effectiveness in the populations.

## Supplementary Information

Supplementary Material 1

## Data Availability

All data and code are available in https://github.com/nordlinglab/COVID19-VaccinePhase3MetaAnalysis.

## Abbreviations

WHO: World Health Organization
EUL: Emergency use listing
CI: Confidence interval
VOC: Variants of Concern
PRISMA: Preferred Reporting Items for Systematic Reviews and Meta-Analyses
