# Supplementary material for: A meta-analysis of vaccine efficacy from phase III clinical trials of approved vaccines against SARS-CoV-2 and variants
Source: BMC Infect Dis. 2025 Sep 26;25:1169. doi: 10.1186/s12879-025-11289-4 (PMC12465431; doi:10.1186/s12879-025-11289-4)
Supplement: Supplementary file 1 — Supplementary Material 1 [file 12879_2025_11289_MOESM1_ESM.pdf]

A meta-analysis of vaccine efficacy from phase III clinical trials of approved vaccines against SARS-CoV-2 and variants

Dipesh Dhayfule†[1], Yu-Heng Wu†[1], Akram Ashyani[1], Ming-Chi Li[2], Chin-Shiang Tsai[2], Po-Lin Chen[2], Torbjörn E. M. Nordling[1*]

[1*]Department of Mechanical Engineeringent, National Cheng Kung University, No. 1 University Rd., Tainan, 701, Taiwan.
[2]Department of Internal Medicine, National Cheng Kung University Hospital, No. 138 Sheng - Li Rd, Tainan, 701, Taiwan.

*Corresponding author(s). E-mail(s): torbjorn.nordling@nordlinglab.org;

The supplementary document contains definition of measure endpoints defined in the articles, method to calculate average vaccine efficacy, vaccines' efficacy comparison, list of clinical studies, list of Variants of Concern (VOC), variants of investigation (VOI), Variants under Monitoring (VUM), and formerly known variants.

†These authors contributed equally to this work.

1 Endpoint definitions

Following are the definitions of endpoints defined in the articles:

Documented Infection against SARS-COV-2 infection - “Laboratory confirmed infections of asymptomatic and symptomatic COVID-19” [1].

Asymptomatic infection - “RT-PCR-confirmed SARS-CoV-2 infection detected from the monthly swabs with none of the pre specified COVID-19 symptoms, reported after receipt of two doses of vaccine or placebo” [2].

Symptomatic COVID-19 confirmed COVID-19 - “One or more symptoms (some articles require minimum two symptoms) that included fever (temperature), chills, new cough, myalgia, headache, sore throat, diarrhoea, nausea, or congestion; or at least one event of new-onset anosmia or ageusia, a respiratory sign or symptom (shortness of breath or difficulty breathing, oxygen saturation <94% or requirement for supplemental oxygen, or radiographic evidence of pneumonia), evidence of shock, or intensive care admission or death along with RT-PCR positive test report” [2].

Mild COVID-19 - “More than one of the following symptoms: fever (defined by subjective or objective measure, regardless of use of anti-pyrectic medications); new onset of cough; more than two additional COVID-19 symptoms: new onset or worsening of shortness of breath or difficulty breathing compared to baseline, new onset of fatigue, new onset of generalized muscle or body aches, new onset of headache, new loss of taste or smell, acute onset of sore throat, congestion, or runny nose, new onset of nausea, vomiting, or diarrhoea” [3].

Moderate COVID-19 - “A positive RT-PCR test for SARS-CoV-2 and two or more of the following symptoms (new or worsening): fever or chills, cough, heart rate

$\geq$90 beats/minute, muscle or body pain, headache, new loss of taste or smell, sore throat, red or bruised-looking feet or toes, nausea, vomiting, or diarrhoea; or one or more of the following signs or symptoms: shortness of breath, respiratory rate $>$20 breaths/minute, clinical or radiologic evidence of pneumonia, deep vein thrombosis, or abnormal oxygen saturation but above 93%" [4].

Severe COVID-19 (without death) - "Rrespiratory rate more than 30/min; Oxygen saturation level $\leq$93%; Oxygen partial pressure/inspiratory oxygen fraction $\leq$300 mmHg; Progression of changes in the lungs according to X-ray, computer tomography (CT), ultrasonography (U/S) (increase in the volume of changes in the lungs by more than 50% after 24-48 hours); Decreased level of consciousness, agitation; Unstable haemodynamics (systolic blood pressure less than 90 mm Hg or diastolic blood pressure less than 60 mm Hg, diuresis less than 20 mL/hr); Arterial blood lactate $>$ 2 mmol/l; More than 2 points on the Sequential Organ Failure Assessment Scale (SOFA) scale" [5].

Hospitalization - "As being admitted due to COVID-19" [6]. Severe COVID-19 (Without death) - "RR (respiratory rate) more than 30/min; Oxygen saturation level $\leq$93%; Oxygen partial pressure/inspiratory oxygen fraction $\leq$300 mmHg; Progression of changes in the lungs according to X-ray, CT, ultrasonography (U/S) (increase in the volume of changes in the lungs by more than 50% after 24-48 hours); Decreased level of consciousness, agitation; Unstable haemodynamics (systolic blood pressure less than 90 mm Hg or diastolic blood pressure less than 60 mm Hg, diuresis less than 20 mL/hr); Arterial blood lactate $>$ 2 mmol/l; More than 2 points on the Sequential Organ Failure Assessment Scale (SOFA) scale" [5].

Emergency department (ED) visits - "The incidence of COVID-19-related Emergency Department visits occurring ≥15 days post second dose of study intervention" [7].

Severe COVID-19 (with death) - "Tachypnea: ≥30 breaths per minute at rest, resting heart rate ≥125 beats per minute, SpO_2: ≤93% on room air or PaO_2/FiO_2<300 mmHg, high flow oxygen (O_2) therapy or non-invasive ventilation (NIV)/non-invasive positive pressure ventilation (NIPPV) (*e.g.*, continuous positive airway pressure [CPAP] or bi-level positive airway pressure [BiPAP]), mechanical ventilation or extracorporeal membrane oxygenation (ECMO), one or more major organ system dysfunction or failure to be defined by diagnostic testing/clinical syndrome/interventions, including any of the following: Acute respiratory failure, including acute respiratory distress syndrome (ARDS), acute renal failure, acute hepatic failure, acute right or left heart failure. Septic or cardiogenic shock (with shock defined as systolic blood pressure [SBP] <90 mm Hg OR diastolic blood pressure [DBP] <60 mm Hg), Acute stroke (ischaemic or haemorrhagic), Acute thrombotic event: acute myocardial infarction (AMI), deep vein thrombosis (DVT), pulmonary embolism (PE), Requirement for: vasopressors, systemic corticosteroids, or haemodialysis, Admission to an intensive care unit (ICU), Death" [8].

Critical COVID-19 (with death) - "A positive RT-PCR test for SARS-CoV-2 with one of the following features: respiratory failure; evidence of shock (systolic blood pressure <90 mm Hg, diastolic blood pressure <60 mm Hg, or requiring vasopressors); respiratory rate >30 breaths/minute; heart rate ≥125 beats/minute; oxygen saturation of 93% or less (ambient air at sea level), or a ratio of the partial pressure of oxygen to

the fraction of inspired oxygen <300 mm Hg; intensive care unit admission; significant acute renal, hepatic, or neurologic dysfunction, or death” [4].

Death - “Death due to COVID-19” [6].

2 Confidence interval calculation methods

We calculated the vaccine efficacy of all endpoint measures to SARS-CoV-2 using the risk ratio. The risk ratio is a multiplicative measure. Taking its natural logarithm transforms the parameter to an additive scale on which the sampling distribution is approximately normal for moderate-to-large counts by standard likelihood theory. This stabilises the variance and yields symmetric confidence limits on the log scale, which translate into asymmetric yet non-negative limits for the risk ratio and, after the transformation $\mathrm{VE} = 1 - R_{\mathrm{RR}}$, for vaccine efficacy. The delta method then shows that $\widehat{\mathrm{Var}}\big[\ln \hat{R}_{\mathrm{RR}}\big]$ is well approximated by the inverse event counts used here, providing a simple closed form for the interval calculation. Denote the standard error, i.e. square root of the variance, by $\widehat{\mathrm{SE}}\big[\ln \hat{R}_{\mathrm{RR}}^{(\mathrm{pool})}\big]$. A two-sided $100(1-\alpha)\%$ confidence interval for the pooled risk ratio is then

$$R_{\mathrm{RR},L} = \hat{R}_{\mathrm{RR}} \exp\Big(-z_{1-\alpha/2}\, \widehat{\mathrm{SE}}\big[\ln \hat{R}_{\mathrm{RR}}\big]\Big), \tag{1}$$

$$R_{\mathrm{RR},U} = \hat{R}_{\mathrm{RR}} \exp\Big(\ z_{1-\alpha/2}\, \widehat{\mathrm{SE}}\big[\ln \hat{R}_{\mathrm{RR}}\big]\Big). \tag{2}$$

Here $z_{1-\alpha/2}$ is the $(1-\alpha/2)$ quantile of the standard normal distribution; for a 95% confidence level, $z_{0.975} = 1.96$. Transforming back to vaccine efficacy,

$$\mathrm{VE}_L^{(\mathrm{pool})} = \big(1 - R_{\mathrm{RR},U}^{(\mathrm{pool})}\big) \times 100\%, \qquad \mathrm{VE}_U^{(\mathrm{pool})} = \big(1 - R_{\mathrm{RR},L}^{(\mathrm{pool})}\big) \times 100\%. \tag{3}$$

2.1 Pooled point estimate

In addition to estimating the vaccine efficacy and confidence intervals of an individual study, we also pool over all studies with the same endpoint. Let x_{ve}, x_{pe} be the total numbers of events (infections) and x_{v}, x_{p} the total participant counts in the vaccine and placebo arms after summing over all trials with the same endpoint. The pooled risk ratio is, analogous to the single study case described in the Methods section,

$$\hat{R}_{\mathrm{RR}}^{(\mathrm{pool})} = \frac{x_{\mathrm{ve}}/x_{\mathrm{v}}}{x_{\mathrm{pe}}/x_{\mathrm{p}}}, \qquad \mathrm{VE}^{(\mathrm{pool})} = \left(1 - \hat{R}_{\mathrm{RR}}^{(\mathrm{pool})}\right) \times 100\%. \tag{4}$$

2.2 Robust variance and confidence interval

Following Zou [10], the sandwich estimate of $\mathrm{Var}\left[\ln \hat{R}_{\mathrm{RR}}^{(\mathrm{pool})}\right]$ is

$$\widehat{\mathrm{Var}}\left[\ln \hat{R}_{\mathrm{RR}}^{(\mathrm{pool})}\right] = \frac{1}{x_{\mathrm{ve}}} - \frac{1}{x_{\mathrm{v}}} + \frac{1}{x_{\mathrm{pe}}} - \frac{1}{x_{\mathrm{p}}}. \tag{5}$$

A two-sided $100(1-\alpha)\%$ confidence interval for the pooled risk ratio is then

$$R_{\mathrm{RR},L}^{(\mathrm{pool})} = \hat{R}_{\mathrm{RR}}^{(\mathrm{pool})} \exp\left(-z_{1-\alpha/2}\, \widehat{\mathrm{SE}}\left[\ln \hat{R}_{\mathrm{RR}}^{(\mathrm{pool})}\right]\right), \tag{6}$$

$$R_{\mathrm{RR},U}^{(\mathrm{pool})} = \hat{R}_{\mathrm{RR}}^{(\mathrm{pool})} \exp\left(z_{1-\alpha/2}\, \widehat{\mathrm{SE}}\left[\ln \hat{R}_{\mathrm{RR}}^{(\mathrm{pool})}\right]\right). \tag{7}$$

Here $z_{1-\alpha/2}$ is the $(1-\alpha/2)$ quantile of the standard normal distribution; for a 95% confidence level, $z_{0.975} = 1.96$. Transforming back to vaccine efficacy,

$$\mathrm{VE}_L^{(\mathrm{pool})} = \big(1 - R_{\mathrm{RR},U}^{(\mathrm{pool})}\big) \times 100\%, \qquad \mathrm{VE}_U^{(\mathrm{pool})} = \big(1 - R_{\mathrm{RR},L}^{(\mathrm{pool})}\big) \times 100\%. \tag{8}$$

2.3 Illustrative calculation for the symptomatic endpoint

Across all trials reporting symptomatic COVID-19, we observed $x_{\mathrm{ve}} = 1092$, $x_{\mathrm{pe}} = 4191$, $x_{\mathrm{v}} = 307{,}902$, $x_{\mathrm{p}} = 264{,}651$. Substituting these totals,

$$\hat{R}_{\mathrm{RR}}^{(\mathrm{pool})} = \frac{1092/307{,}902}{4191/264{,}651} \;=\; 0.224, \tag{9}$$

$$\widehat{\mathrm{VE}}^{(\mathrm{pool})} = (1 - 0.224) \times 100\% \;=\; 77.60\%. \tag{10}$$

The robust standard error is

$$\widehat{\mathrm{SE}}\big[\ln \hat{R}_{\mathrm{RR}}^{(\mathrm{pool})}\big] = \sqrt{\tfrac{1}{1092} - \tfrac{1}{307{,}902} + \tfrac{1}{4191} - \tfrac{1}{264{,}651}} \;=\; 0.0339. \tag{11}$$

Consequently, the 95% confidence limits for the pooled VE are

$$\mathrm{VE}_L^{(\mathrm{pool})} = 76.06\%, \qquad \mathrm{VE}_U^{(\mathrm{pool})} = 79.04\%. \tag{12}$$

In other words, the vaccine efficacy of all vaccines against symptomatic endpoint is 77.60% (95% CI 76.06-79.04%).

3 Vaccines' efficacy comparison

Zero events in any group make the standard deviation of the relative risk undefined due to division by zero. To avoid division by zero it is common to add 0.5 to the elements of the contingency table (see [11]) Figure 1 is the same as Figure 3 in the main text but with the 0.5 addition to the contingency table when there is at least one 0 in the table.

Figure 2 and 3 compare the reproduced vaccine efficacy and its confidence interval estimated using relative risk and Poisson regression with robust error variance with the vaccination efficacy and its confidence interval from the studies. The chart makes it obvious that the majority of the studies' endpoints for vaccination efficacy and its confidence interval agree with our reproduction of vaccine efficacy and its confidence interval. Additionally, we discovered that more than 95% of endpoints had vaccine efficacy of at least 60% against the SARS-CoV-2 and its variant. The biggest disparity between the original vaccine efficacy and the reproduced vaccine efficacy that we identified is for the endpoint moderate in [12] with a difference of 19.5% between the calculated and reproduced vaccine efficacy. The authors of [12] uses the hazard ratio to calculate vaccine efficacy and the Clopper-Pearson method to calculate confidence intervals in the study. The endpoint shows vaccine efficacy of 83.7% (95% CI, 58-93,7) against moderate SARS-CoV-2 using CoronaVac in phase III clinical trial.

The 0.5 addition typically generates more conservative (smaller) lower bounds when $x_{\text{ve}} = 0$. Figures 4 and 5 replicates Figure 4 and 5 in the main text but include data points with zero events that have been adjusted using the 0.5 addition method.

The different vaccine efficacy and confidence interval estimation methods are compared with our reproduction, and the differences in vaccine efficacy are shown in Figure 6. Since some confidence interval methods produce unequal lengths from the efficacy to the lower and upper bounds, we compare both cases in Figures 9 and 11. If the original study did not provide the confidence interval or the infected population in the vaccine group, the data were not considered in the analysis of the confidence interval.

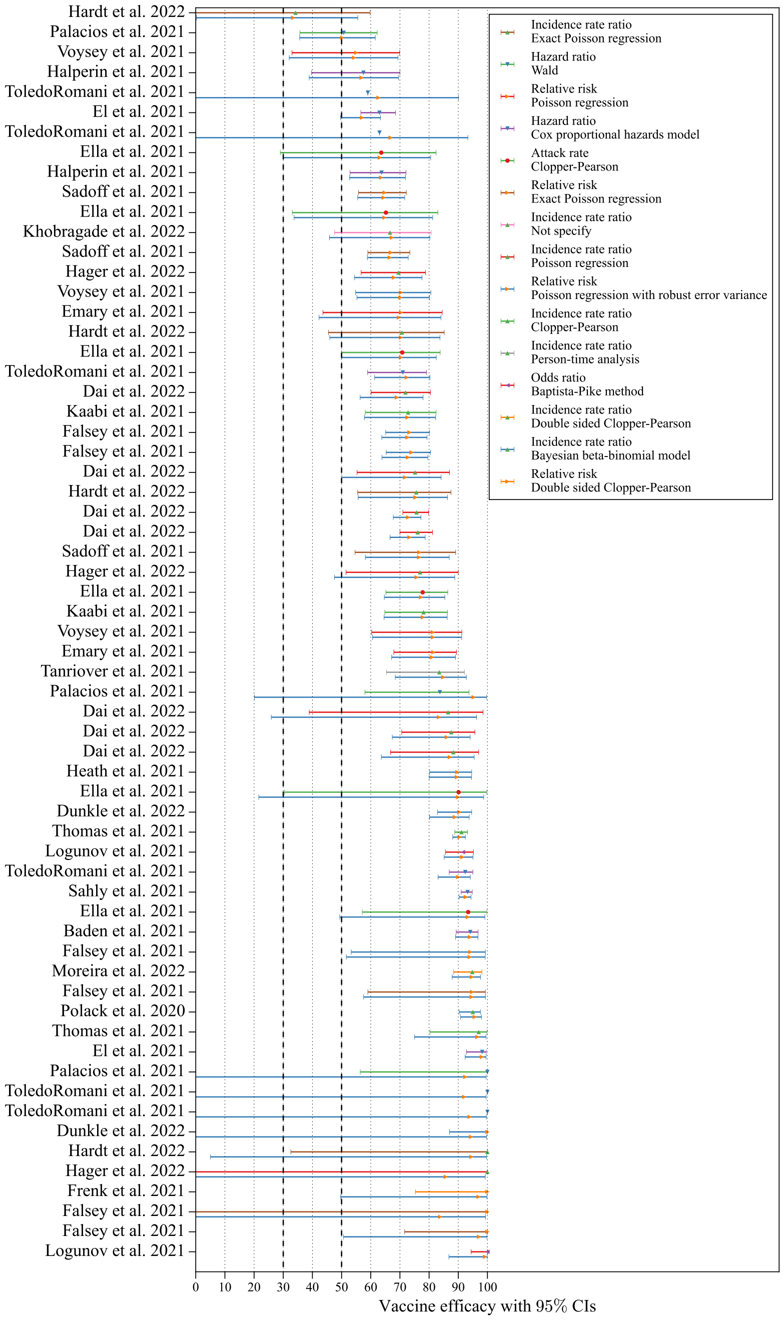

Fig. 1: Vaccine efficacy and its confidence interval from the studies (the first line in each study) compared against the reproduced results (the second line in each study). The vaccine efficacies and its confidence intervals are reproduced using relative risk and Poisson regression with robust error variance. The 0.5 adjustment has been applied.

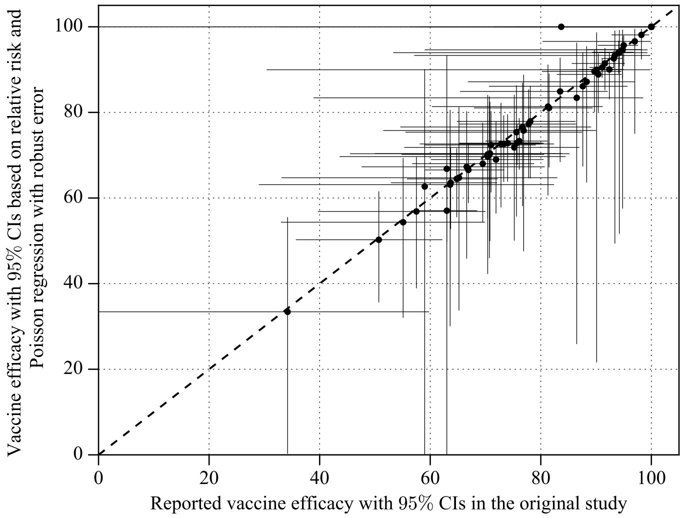

Fig. 2: Scatter plot of the comparison of reproduced vaccine efficacy and the original study. The relative risk and Poisson regression with robust error are used for reproduction.

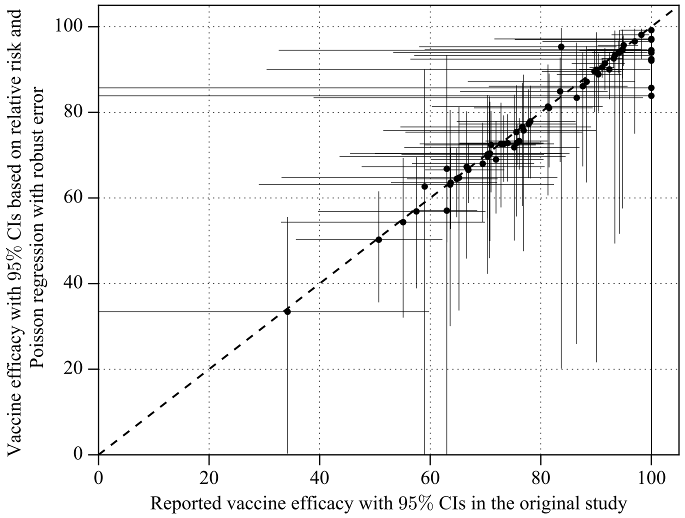

Fig. 3: Scatter plot of the comparison of reproduced vaccine efficacy and the original study. The relative risk and Poisson regression with robust error are used for reproduction. The 0.5 adjustment has been applied.

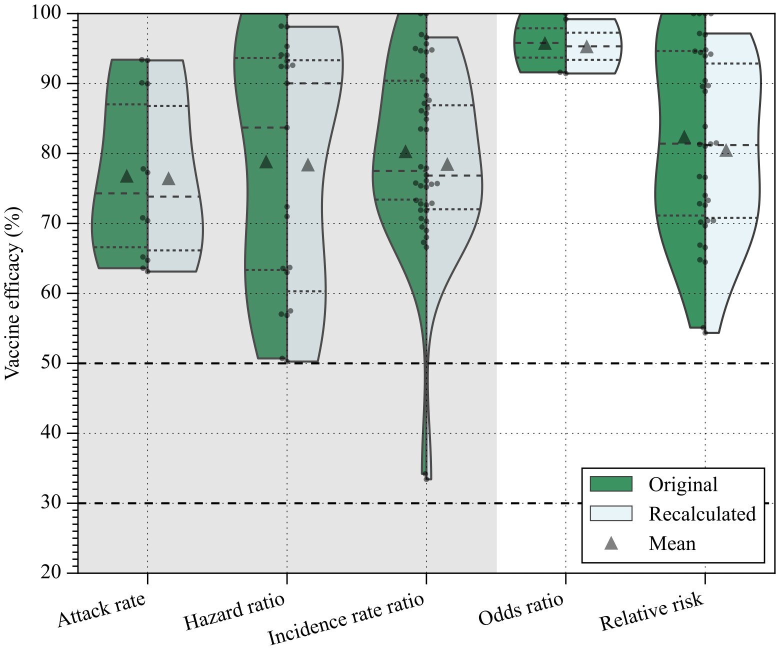

Fig. 4: Violin plot illustrating the comparison of the original vaccine efficacy vs our recalculation using the relative risk method. The mean is marked with a triangle, and each dot represents one data point. The dashed lines in the violin plot indicate the first, second, and third quartiles. The original methods utilizing time-to-event data are indicated by the shaded area. The critical thresholds of 50% and 30% are indicated by the dashed lines.The 0.5 adjustment has been applied.

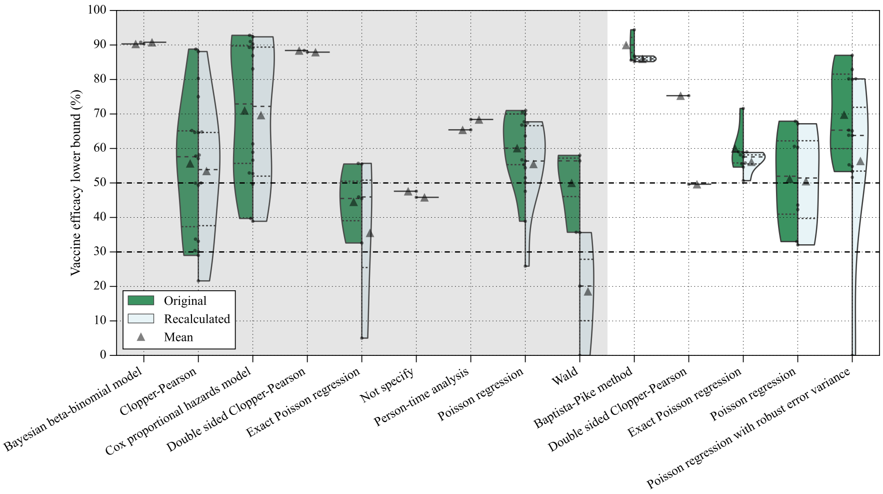

Fig. 5: Violin plot illustrating the comparison of the original vaccine efficacy 95% CI lower bound vs our recalculation using the Poisson regression with robust error method. The mean is marked with a triangle, and each dot represents one data point. The dashed lines in the violin plot indicate the first, second, and third quartiles. The original methods utilizing time-to-event data are indicated by the shaded area. The critical thresholds of 50% and 30% are indicated by the dashed lines. The 0.5 adjustment has been applied.

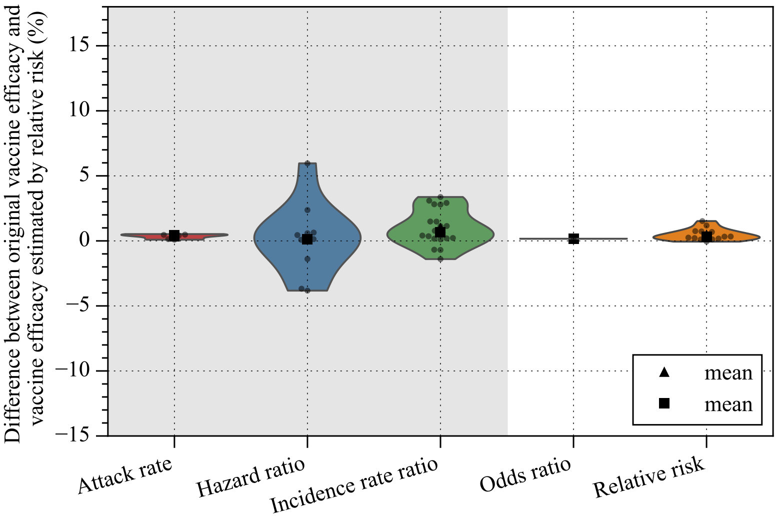

Fig. 6: Violin plot illustrating the distances between reported vaccine efficacies and our recalculated values using the relative risk method. The original methods utilizing time-to-event data are indicated by the shaded area. The positive value indicates that the reported vaccine efficacy is higher than the calculated value.

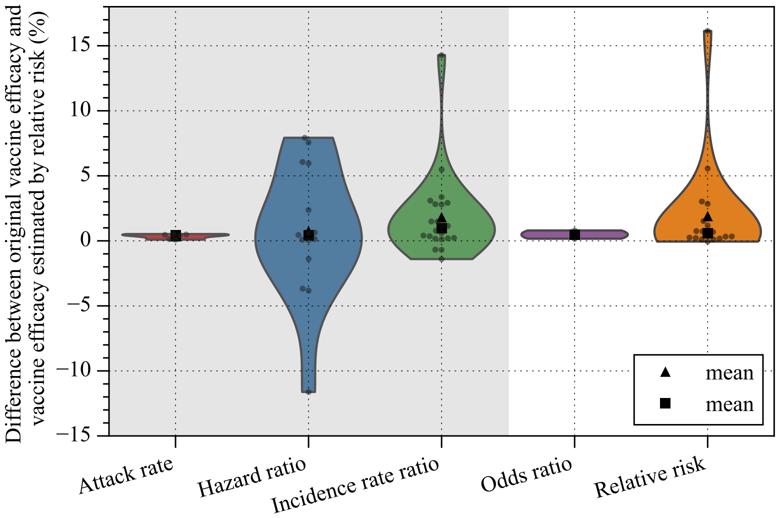

Fig. 7: Violin plot illustrating the distances between reported vaccine efficacies and our recalculated values using the relative risk method. The original methods utilizing time-to-event data are indicated by the shaded area. The positive value indicates that the reported vaccine efficacy is higher than the calculated value. The 0.5 adjustment has been applied.

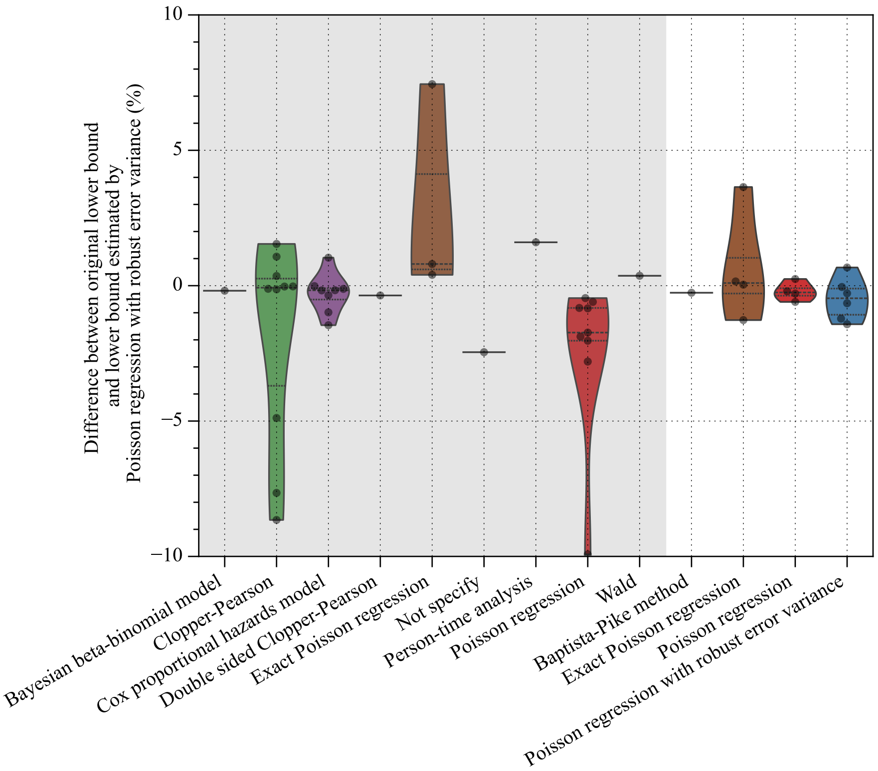

Fig. 8: Violin plot of the differences between the reported lower bound distances and our recalculation using the Poisson regression with robust error. The original methods utilizing time-to-event data are indicated by the shaded area.

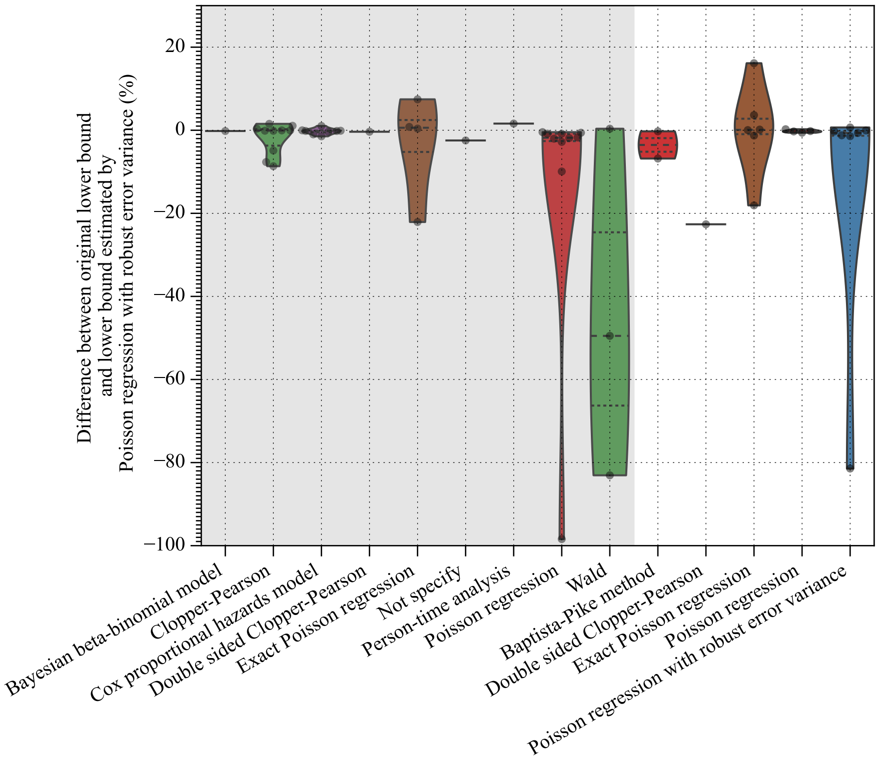

Fig. 9: Violin plot of the differences between the reported lower bound distances and our recalculation using the Poisson regression with robust error. The original methods utilizing time-to-event data are indicated by the shaded area. The 0.5 adjustment has been applied.

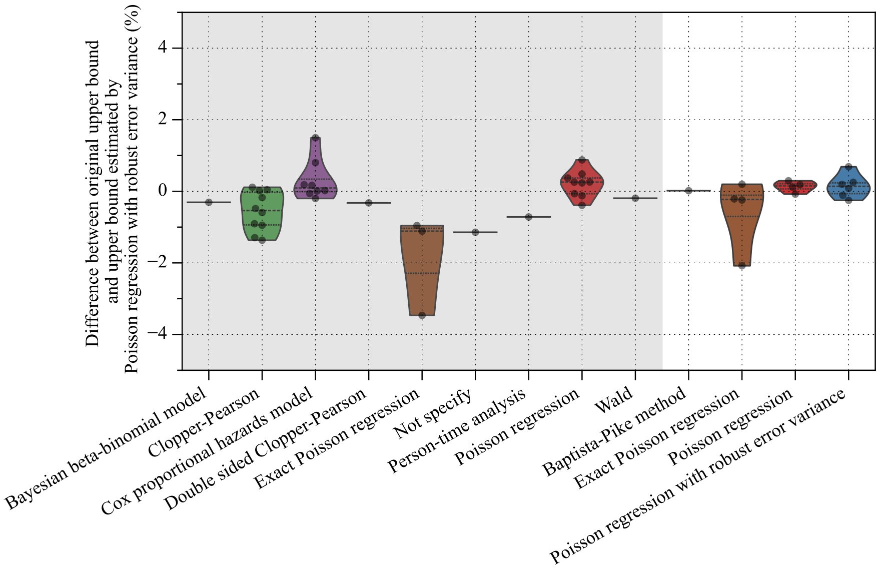

Fig. 10: Violin plot of the differences between the reported upper bound distances and our recalculation using the Poisson regression with robust error. The original methods utilizing time-to-event data are indicated by the shaded area.

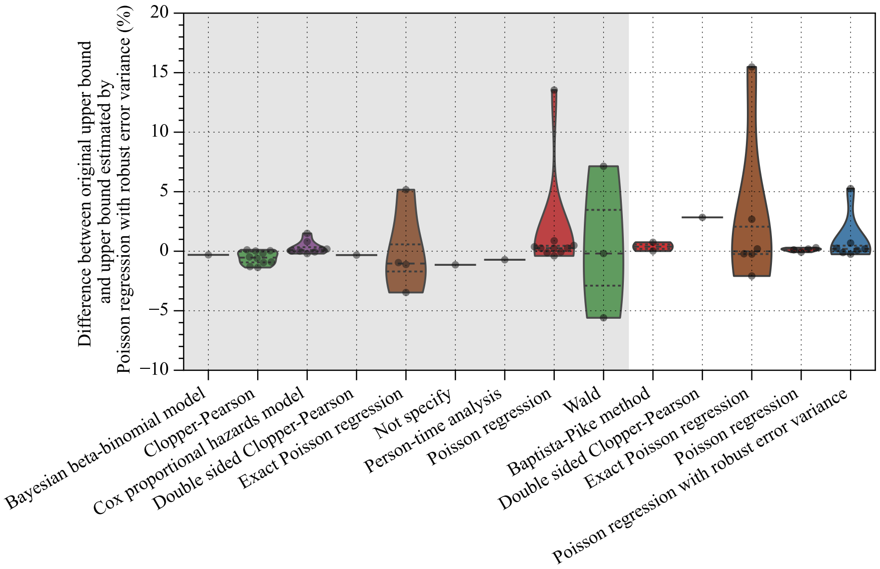

Fig. 11: Violin plot of the differences between the reported upper bound distances and our recalculation using the Poisson regression with robust error. The original methods utilizing time-to-event data are indicated by the shaded area. The 0.5 adjustment has been applied.

4 Publication bias

Funnel plots provide a simple visualization tool for assessing publication bias in meta-analyses, though they can be misleading when analyzing too few studies. Statistical tests for funnel plot asymmetry require at least 10 studies to achieve adequate power [13], making analysis of scenarios with fewer data points less meaningful. We first tallied the available data points in our studies by endpoint, variant, and vaccine, as shown in Tables 1 to 10. Only studies examining vaccines against the symptomatic endpoint of the original SARS-CoV-2 variant contained more than 10 data points. The funnel plots for assessing publication bias are presented in Figure 12, including plots for both the symptomatic endpoint and the severe+ category (comprising severe, critical, death, ED, and ICU). Egger's test revealed significant asymmetry for the symptomatic endpoint but not for the severe+ endpoint. To examine the impact of large variance cases (small sample size) in the symptomatic endpoint, we excluded the Frenck2021 study [14]. The resulting funnel plot, shown in Figure 13 and Table 11, demonstrates only minor changes (0.01 differences in both Fixed Effects and Random Effects) compared to the left plot in Figure 12 and Table 4 in the main text.

Table 1: Data counts for asymptomatic endpoint.

Vaccine	SARS-CoV-2	Total
Ad26.COV2.S*	1	1
BBV152	1	1
mRNA1273	1	1
Total	3	3

Table 2: Data counts for critical endpoint.

Vaccine	SARS-CoV-2	Total
AZD1222	1	1
Ad26.COV2.S	1	1
Ad26.COV2.S*	1	1
ZF2001	1	1
Total	4	4

Table 3: Data counts for death endpoint.

Vaccine	SARS-CoV-2	Total
SOBERANA Plus	1	1
SOBERANA-02	1	1
ZF2001	1	1
Total	3	3

Table 4: Data counts for documented infection endpoint.

Vaccine	SARS-CoV-2	Total
BNT162b2	1	1
Total	1	1

Table 5: Data counts for ED endpoint.

Vaccine	SARS-CoV-2	Total
AZD1222	1	1
Total	1	1

Table 6: Data counts for hospitalization endpoint.

Vaccine	SARS-CoV-2	Total
AZD1222	1	1
Total	1	1

Table 7: Data counts for icu endpoint.

Vaccine	SARS-CoV-2	Total
AZD1222	1	1
Total	1	1

Table 8: Data counts for moderate endpoint.

Vaccine	SARS-CoV-2	Total
Ad26.COV2.S	1	1
Ad26.COV2.S*	1	1
CoVLP	1	1
CoronaVac	1	1
Total	4	4

Table 9: Data counts for severe endpoint.

Vaccine	SARS-CoV-2	Total
BBV152	1	1
BNT162b2	1	1
CoVLP	1	1
CoronaVac	1	1
Novavax	1	1
SOBERANA Plus	1	1
SOBERANA-02	1	1
Sputnik V	1	1
mRNA1273	1	1
Total	9	9

Table 10: Data counts for symptomatic endpoint.

Vaccine	Alpha	Beta	Delta	Kappa	Mixed	Non beta	SARS-CoV-2	Total
AZD1222	0	1	0	0	0	1	3	5
AZD1222*	0	0	0	0	0	0	2	2
Ad26.COV2.S	0	0	0	0	0	0	1	1
Ad26.COV2.S*	0	0	0	0	0	0	1	1
Ad5-nCOV	0	0	0	0	0	0	2	2
BBIBP-CorV	0	0	0	0	0	0	1	1
BBV152	0	0	1	1	1	0	1	4
BNT162b2	0	0	0	0	0	0	2	2
BNT162b2*	0	0	0	0	1	0	0	1
CoVLP	0	0	0	0	0	0	1	1
CoronaVac	0	0	0	0	0	0	2	2
Novavax	0	0	0	0	0	0	2	2
SOBERANA Plus	0	0	0	0	0	0	1	1
SOBERANA-02	0	0	0	0	0	0	1	1
Sputnik V	0	0	0	0	0	0	1	1
Vero cells	0	0	0	0	0	0	1	1
ZF2001	1	0	1	1	1	0	1	5
ZyCov-D	0	0	0	0	1	0	0	1
mRNA1273	0	0	0	0	0	0	2	2
Total	1	1	2	2	4	1	**25**	36

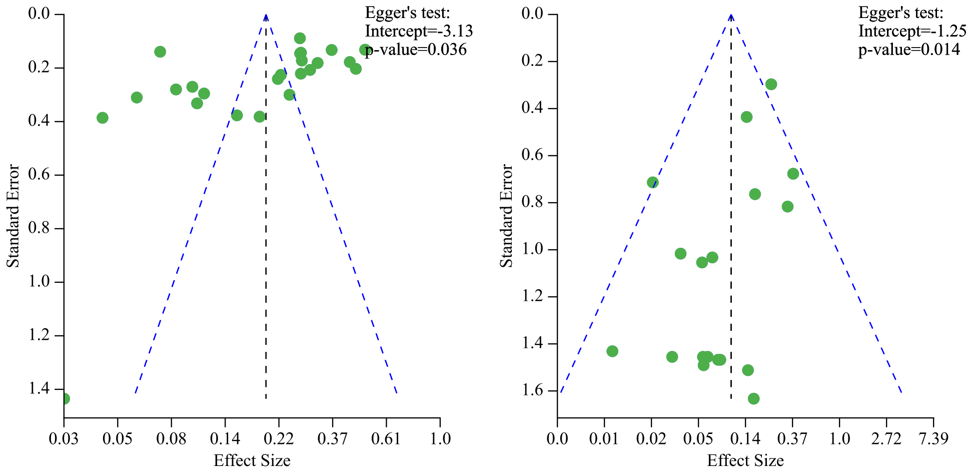

Fig. 12: Figure shows funnel plots of vaccine efficacy risk ratios derived from random effects models. The left panel represents efficacy against symptomatic endpoint in SARS-CoV-2, while the right panel shows efficacy against severe+ (severe, critical, death, ED, and ICU) in the original SARS-CoV-2 study.

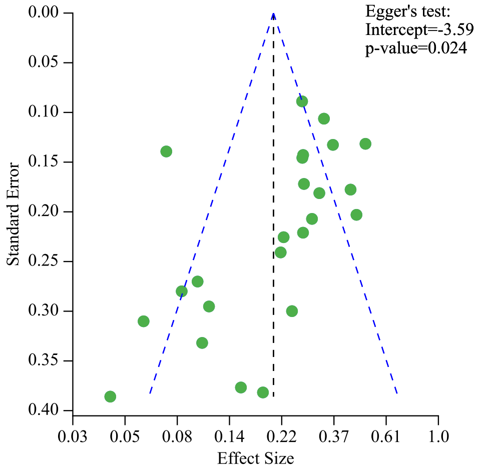

Fig. 13: The funnel plot of vaccine efficacy against symptomic endpoint in SARS-CoV-2 with one small size sample (Frenck2021 [14]) dropped. The risk ratios are derived from random effects models.

Table 11: Summary of meta-analysis of vaccine efficacy for SARS-CoV-2 symptomatic endpoint with small sample (Frenck2021 [14]) dropped, including Risk Ratio (RR) with 95% Confidence Intervals, weights of fixed effects (FE), random effects (RE), and meta-analysis summary statistics.

Vaccine name [ref]	RR (95%CI)	FE (%)	RE (%)
AZD1222 [15]	0.30 (0.20–0.45)	2.41	4.31
AZD1222 [7]	0.27 (0.20–0.36)	4.19	4.59
AZD1222 [7]	0.27 (0.21–0.36)	4.34	4.60
AZD1222* [15]	0.46 (0.31–0.68)	1.82	4.33
AZD1222* [15]	0.19 (0.09–0.39)	1.05	3.32
Ad26.COV2.S [4]	0.33 (0.27–0.41)	8.38	4.74
Ad26.COV2.S* [16]	0.25 (0.14–0.44)	1.31	3.79
Ad5-nCOV [17]	0.43 (0.30–0.61)	2.50	4.45
Ad5-nCOV [17]	0.36 (0.28–0.47)	5.05	4.64
BBIBP-CorV [18]	0.22 (0.14–0.35)	2.27	4.13
BBV152 [2]	0.23 (0.15–0.35)	2.53	4.21
BNT162b2 [19]	0.04 (0.02–0.09)	3.87	3.30
CoVLP [20]	0.32 (0.22–0.46)	2.99	4.43
CoronaVac [21]	0.15 (0.07–0.32)	1.00	3.35
CoronaVac [12]	0.50 (0.38–0.64)	4.05	4.65
Novavax [8]	0.10 (0.05–0.20)	2.29	3.61
Novavax [3]	0.11 (0.06–0.20)	2.01	3.82
SOBERANAPlus [22]	0.10 (0.06–0.17)	3.65	3.96
SOBERANA-02 [22]	0.28 (0.20–0.39)	3.71	4.48
SputnikV [5]	0.09 (0.05–0.15)	2.22	3.91
Verocells [18]	0.27 (0.18–0.42)	2.27	4.23
ZF2001 [23]	0.27 (0.23–0.32)	13.89	4.79
mRNA1273 [24]	0.07 (0.06–0.10)	17.78	4.62
mRNA1273 [25]	0.06 (0.03–0.11)	4.42	3.73
Total		Fix	Rand
Fixed Effects	0.22 (0.21–0.24)		
Random Effects	0.21 (0.16–0.26)		

Heterogeneity measures:
$\tau^2 = 0.304$
$I^2 = 90.38\%$
Q(Chi-square)=239.08 ($p \leq 0.001$)
Overall effect test: z=42.24 (Fixed), z=12.91 (Random), $p \leq 0.001$
Egger's test: t=-2.42, p=0.024

5 Data available

We looked into the sources of funding for phase III clinical trials as well as the types of data that were made publically accessible for additional clinical trial analysis. Table 12 contains the author's name, the targeted vaccine, the source of trial funding, clinical trial, the type of publicly available data, and how anyone can access it. We divide the data into four categories and check with each article what type of data is exactly available for the readers. The four categories of the data are: A) Only summary statistics (*e.g.*, efficacy and CI). B) Counts of number of subjects in each possible outcome (e.g., number infected, number not infected, number vaccinated, and number placebo). C) Subject characteristics without any personally identifying data (e.g., age, gender, enrolment date, and event record with dates). D) Raw data (i.e., all original reports). All the columns are marked with "I (Included)", "O (On request)", "N (Not mentioned)", and "P (Post publication)".

6 Vaccine description

Out of 38 vaccines, 15 vaccines were developed on a protein subunit platform, 12 on an inactivated platform, 6 on a non-replicating viral vector platform, 3 on an RNA platform and 1 vaccine each on VLP, DNA, and recombinant protein subunit platforms. Oxford Univ./AstraZeneca was the first Research and Development institute which announce its Phase I clinical trials for the potential vaccine candidate ChAdOx1 nCov-19 Vaccine (AZD1222) popularly also known as "Vaxzevria". The Serum Institute of India's "Covishield" vaccine is a daughter vaccine of the AZD1222 parent

vaccine. Covishield vaccine is distributed in two doses per individual in 28-day intervals, has a shelf life of six months, and can be stored at 2 to 8°C for six months [30]. The vaccine is approved and available to be used in 140 countries with 63 clinical trials conducted in 33 countries [31]. On 24th November 2020, Russia's National Research Centre for Epidemiology and Microbiology announced Gam-Covid-Vac known as the "Sputnik V" vaccine developed on a non-replicating viral vector platform developed by Gamaleya Institute. The vaccine is stored at -18°C and has a shelf life of 6 months [5]. Sputnik V is available in 74 countries and has had 24 clinical trials conducted in 7 countries [31]. The vaccine developed by Pfizer/BioNTech BNT162b2 is an mRNA-based vaccine available in 146 countries with 80 clinical trials in 26 countries [31]. The vaccine can be stored at -80 to -60°C for 6 months and can be stored at a refrigerator temperature of 2 to 8°C for 1 month [32]. Moderna's mRNA-1273 is built on an RNA platform that requires a storage temperature of -20°C [25]. The vaccine has undergone 63 clinical trials in 22 countries till now and has been approved for use in 86 countries [31]. Takeda-919 is the daughter vaccine of the mRNA-1273 parent vaccine. Ad26.COV2S, also known as Janssen, is the first one-dose vaccine developed by Johnsson & Johnsson against SARS-CoV-2. Currently, the vaccine is used in 108 countries [31]. The vaccine must be stored at a temperature of -25 to -15°C. JnJ has carried out 23 clinical trials in 23 countries [4, 31]. The remaining one-dose vaccines are Sputnik Light, Soberana Plus, and Adn5COV. The Novavax NVX- is a protein subunit vaccine which is approved for use in 37 countries. The Covovax vaccine is the daughter vaccine of Novavax vaccine. Three vaccine (CIGB-66, Razi COV Pars and ZyCOV-D) requires 3-dose vaccine against the novel coronavirus [31].

ZyCoV-D, manufactured by Zycov Cadila, has the best temperature storage and transportation at 30°C, and out of the remaining vaccines, 21 vaccines can be stored at 2-8°C. Ad26. COV2S and Covilo vaccines have a maximum shelf life of 24 months after manufacturing. Seven vaccines have received approval from more than 50 countries. Thirteen vaccines only received approval from 1 country for usage. CoronaVac, Ad5-nCOV, and inactivated vaccine all have a 12-month shelf life before expiration [31]. We identified four-pairs of parent-daughter vaccine 1) mRNA-1273 - Takeda-919^{D}, 2)AZD1222 - Covishield^{D}, 3) Novavax - Covovax^{D}, and 4) Novavax - Tak-019^{D} which shares the chemical formulation.

7 Variants of Concern

Table 13 displays the 1 Variant of Concern (VOC) and 4 previously circulating VOCs declared by WHO and the newly released labels along with their Pango lineages for all the variants on 18th December 2021. "A variant is classified as VOC if it satisfies some or all of the criteria such as increased transmissibility, morbidity, mortality, risk of post covid syndrome, affinity for particular demographic or clinical group, ability to evade detection by diagnostic test, decreased susceptibility to antiviral drugs, neutralizing bodies, and ability to cause reinfection" [33, 34]. The whole-genome sequencing technique is used to detect the type of variants from the patient's nasal swab samples [35].

8 Previously circulating COVID-19 Variants of Interest

"Variants of Interest have the genetic mutations associated with changes in receptor binding, lower neutralization by antibodies developed against previous infection or vaccination, reduced treatment efficacy, potential diagnostic impact, or projected increase in transmissibility or disease severity" [44]. WHO amends the list of Variants of Interest (VOI) regularly, now they are termed as Previously circulating VOIs as described in Table 14.

On regular basis WHO updates the list of VOC and VOI based on the transmission and severity of the variants. Variants under monitoring are those that are circulating but whose biological properties have yet to be established [31]. "Former VOCs/VOIs/VUMs, including their descendent lineages, that have been reclassified based on at least one the following criteria":

i) "the variant is no longer circulating at levels of global public health significance"
ii) "the variant has been circulating for a long time without any impact on the overall epidemiological situation"
iii) "scientific evidence demonstrates that the variant is not associated with any concerning properties."

Table 12: List of the clinical studies with funding sources and data availability categorized: A) Only summary statistics (e.g., efficacy and CI). B) Counts of number of subjects in each possible outcome (e.g., number infected, number not infected, number vaccinated, and number placebo). C) Subject characteristics without any personally identifying data (e.g., age, gender, enrolment date, and event record with dates). D) Raw data (i.e., all original reports). Abbreviation, BD —Booster dose, PP III —Post phase III, PC —Prospective Cohort, I —Included, O —On request, N —Not mentioned and P —Post publication. (Updated to 2022-06-16).

Article reference	Target vaccine	Funding source	Study type	A	B	C	D	URL/ Step for data access
[15]	AZD1222	UK Research and Innovation, National Institutes for Health Research (NIHR), Coalition for Epidemic Preparedness Innovations, Bill & Melinda Gates Foundation, Lemann Foundation, Rede D'Or, Brava and Telles Foundation, NIHR Oxford Biomedical Research Centre, Thames Valley and South Midland's NIHR Clinical Research Network, & AstraZeneca	Phase III	I	I	O	O	Email to corresponding author
[7]	AZD1222	AstraZeneca	Phase III	I	I	O	N	https://astrazenecagrouptrials.pharmacm.com/ST/Submission/Disclosure
[26]	AZD1222	UK Research and Innovation, National Institutes of Health Research, Coalition for Epidemic Preparedness Innovations, the Bill & Melinda Gates Foundation, Lemann Foundation, Rede D'Or, the Brava and Telles Foundation, NIHR Oxford Biomedical Research Centre, Thames Valley and South Midland's NIHR Clinical Research Network, and AstraZeneca	PP III	I	I	O	O	Email to corresponding author
[15]	AZD1222*	UK Research and Innovation, National Institutes of Health Research, Coalition for Epidemic Preparedness Innovations, the Bill & Melinda Gates Foundation, Lemann Foundation, Rede D'Or, the Brava and Telles Foundation, NIHR Oxford Biomedical Research Centre, Thames Valley and South Midland's NIHR Clinical Research Network, and AstraZeneca	BD	I	I	O	N	Email to corresponding author
[4]	Ad26.COV2.S	Biomedical Advanced Research and Development Authority, Janssen R&D	Phase III	I	I	O	O	https://www.janssen.com/clinical-trials/transparency
[16]	Ad26.COV2.S*	Biomedical advanced R&D authority, Health and Human Services, National Institute of allergy and infectious disease	BD	I	I	O	O	http://yoda.yale.edu/
[17]	Ad5-nCOV	CanSino Biologics and Beijing Institute of Biotechnology	Phase III	I	I	O	O	Email to corresponding author
[18]	BBIBP-CorV	China National Biotec	Phase III	I	I	N	N	-
[2]	BBV152	Bharat Biotech & Indian Council of Medical Research	Phase III	I	I	O	N	Email to corresponding author
[19]	BNT162b2	BioNTech & Pfizer	Phase III	I	I	O	N	https://www.pfizer.com/science/clinical-trials/data-and-results
[14]	BNT162b2	BioNTech and Pfizer	Phase III (12-15 year old)	I	I	O	N	https://www.pfizer.com/science/clinical-trials/data-and-results
[6]	BNT162b2	None	PC	I	I	N	N	-
[27]	BNT162b2*	BioNtech & Pfizer	BD	I	I	O	N	https://www.pfizer.com/science/clinical-trials/data-and-results
[12]	Coronavac	Fundacao Butantan, Instituto Butantan, and Sao Paulo Research Foundation - FAPESP	Phase III	I	I	N	N	-
[21]	CoronaVac	Health Institutes of Turkey	Phase III	I	I	O	O	Email to corresponding author
[20]	CoVLP	Canadian Innovation, Science & Economic Development Strategic Innovation Fund	Phase III	I	I	O	N	Email to corresponding author
[28]	CVnCOV	German Federal Ministry of Education & Research and CureVac	Phase III	I	I	O	O	Email to corresponding author
[8]	Novavax	Novavax	Phase III	I	I	O	O	https://www.clinicaltrialsregister.eu/ctr-search/trial/2020-004123-16/GB
[3]	Novavax	Novavax	Phase III	I	I	I	I	https://clinicaltrials.gov/ct2/show/NCT04611802
[25]	mRNA-1273	Biomedical Advanced R&D Authority and National Institute of Allergy & Infectious Diseases	Phase III	I	I	O	N	Email to corresponding author
[24]	mRNA-1273	Biomedical Advanced R&D Authority and National Institute of Allergy & Infectious Diseases	Phase IV	I	I	O	O	Email to corresponding author
[22]	Soberana 02	Finlay Vaccine Institute, BioCubaFarma and Fondo Nacional de Cienciay Tecnica	Phase III	I	I	P	P	-
[22]	Soberana Plus	Finlay Vaccine Institute, BioCubaFarma and Fondo Nacional de Cienciay Tecnica	BD	I	I	P	P	-
[5]	Sputnik V	Moscow City Health Department, Russian Direct Investment Fund, and Sberbank	Phase III	I	I	O	O	Email to corresponding author
[18]	Vero cells	China National Biotec	Phase III	I	I	N	N	-
[23]	ZF2001	National Science and Technology, National Natural Science Foundation of China, Chinese Academy of Sciences and Anhui Zhifei Longcom Biopharmaceutical	Phase III	I	I	O	O	Email to corresponding author
[29]	ZyCOV-D	GOI and Cadillac healthcare	Phase III	I	I	O	N	Email to corresponding author

Table 13: Variants of Concern, The VOC along with their Pango lineage and WHO label arranged in Greek alphabetical order. The number of mutations represents the changes in the spike protein including deletion. The * represents designated previously circulating VOC. Abbreviation, NM —Number of mutations; NCC —Number of Countries with Cases detected. (Updated to 2022-06-16).

Pango lineage	WHO label	Date of designation	Country 1st case	Date of detection	Transmission rate (95% CI)	Death rate (95% CI)	NM	Susceptible species	NCC	References
B.1.1.529	Omicron	VUM: 2021-11-24; VOC: 2021-11-26	Multiple	2021-Nov	-	-	60	Human	46	[36]
B.1.1.7	Alpha*	2020-12-18	United Kingdom	2020-Sep	43-90% (38% to 130%)	1.64 (1.32 to 2.04)	23	Human	114	[37]
			Nigeria		150%<br>75 % (70-80%)					[38]
B.1.351	Beta*	2020-12-18	South Africa	2020-Oct	50% (20% to 113%)	1.3	11	Human, Mice	103	[39] [40]
P.1	Gamma*	2021-01-11	Brazil	2020-Dec	250% (230% to 280%)	1.8	17	Human, Mice	71	[41]
					200 % (170% to 240%)					[42]
B1.617.2	Delta*	VOI: 2021-04-04; VOC: 2021-05-11	India	2020-Oct	225 %	-	13	Human	179	[43]

Table 14: Previously circulating VOIs, The VOI along with their Pango lineage and WHO label. Abbreviation, NCC —Number of Countries with Cases detected. (Updated to 2022-06-16).

Pango lineage	WHO label	Date of designation	Country 1st case reported	Date of detection	References
C.37	Lambda	2021-06-14	Peru	2020-Aug	[45]
B.1.621	Mu	2021-08-30	Columbia	2021-Jan	[45]
B1.427; B1.429	Epsilon	VOI: 2021-03-05; Alert: 2021-07-06; Reclassified:2021-11-09	USA	2020-Mar	[45]
P.2	Zeta	VOI: 2021-03-17; VUM: 2021-07-06; Reclassified:2021-08-17	Brazil	2020-Apr	[45]
P.3	Theta	VOI: 2021-03-24; Alert: 2021-07-06; Reclassified:2021-08-17	Philippines	2021-Jan	[45]
B.1.617.1	Kappa	VOI: 2021-04-04, VUM:2021-09-20	India	2020-Oct	[45]
B.125	Eta	VOI: 2021-03-17	Multiple countries	2020-Dec	[45]
B.126	Iota	VOI: 2021-03-17	Multiple countries	2020-Dec	[45]

8.1 Formerly monitored variants

Former VOCs/VOIs/VUMs (Table 15), including their descendent lineages, have been reclassified based on at least one the following criteria:

i) "The variant is no longer circulating at levels of global public health significance"

ii) "The variant has been circulating for a long time without any impact on the overall epidemiological situation"

iii) "scientific evidence demonstrates that the variant is not associated with any concerning properties."

9 Efficacy of Vaccines in Clinical Trials

We retrieved three endpoints of vaccine efficacy against SARS-CoV-2 asymptomatic represents in figure 14: vaccine with BBV152 reporting lower bound of 95% CI closed to 30% with vaccine efficacy 63.1% and its 95% CI, 30-80.5% [2] and mRNA-1273 with vaccine efficacy of 57.0% and 95% CI, 49.1-63.3% [24]. The average vaccine efficacy for asymptomatic is 55.66% (95% CI, 48.76-61.63%) using BBV152 and mRNA-1273 vaccine against SARS-CoV-2.

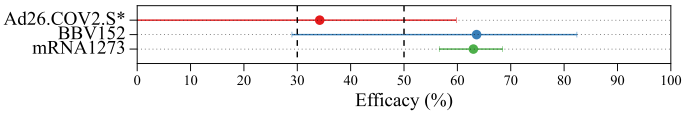

Fig. 14: Vaccine efficacy against SARS-CoV-2 asymptomatic with 95% confidence interval.

Figure 15 is the same as the main text Figure 6 but with the 0.5 method.

Table 15: Formerly monitored Variants, the table represents formerly known VOC and VOI variants, reclassified after assessing the variants as they no longer possess the threat. (Update to 2022-06-17).

Pango lineage	GISAID clade	Next strain clade	Date of designation	Country 1st case reported	References
R.1; R.2	GR	-	VUM: 2021-04-07; Reclassified:2021-11-09	Multiple countries; 2021-Jan	[46]
B.1.466.2	GH	-	VUM:2021-04-28; Reclassified:2021-11-09	Indonesia; 2021-Jan	[45]
B1.620	G	-	VUM:2021-07-14; Reclassified:2021-11-09	Multiple countries; 2020-Nov	[45]
B1.619	G	20A/S.126A	VUM:2021-07-14; Reclassified:2021-11-09	Multiple countries; 2020-May	[45]
AV.1	GR	-	VUM:2021-05-26; Reclassified:2021-07-21	UK; 2021-Mar	[45]
B.1.523	GR	-	VUM:2021-07-14; Reclassified:2021-11-09	Multiple countries; 2021-Jan	[45]
B.1.1.519	GR	20B	VUM:2021-06-02; Reclassified:2021-11-09	Multiple countries; 2020-Nov	[45]
AT.1	GR	-	VUM:2021-06-09, Reclassified:2021-07-21	Russia; 2021-Jan	[45]
C.36.3; C.36.3.1	GR	20D	VUM:2021-06-16; Reclassified:2021-11-09	Multiple countries; 2021-Jan	[45]
B.1.214.2	G	-	VUM:2021-06-30; Reclassified:2021-11-09	Multiple countries; 2020-Nov	[45]
C.1.2	GR	-	2021-09-01	South Africa; 2021-May	[45]
B.1.640	GH/490R	-	2021-11-22	DRC, 2021-Sep	[45]

In the phase 3 clinical trials as shown in the figure 17, four vaccines demonstrated vaccine efficacy against moderate SARS-CoV-2. Coronavac reported 100% vaccine

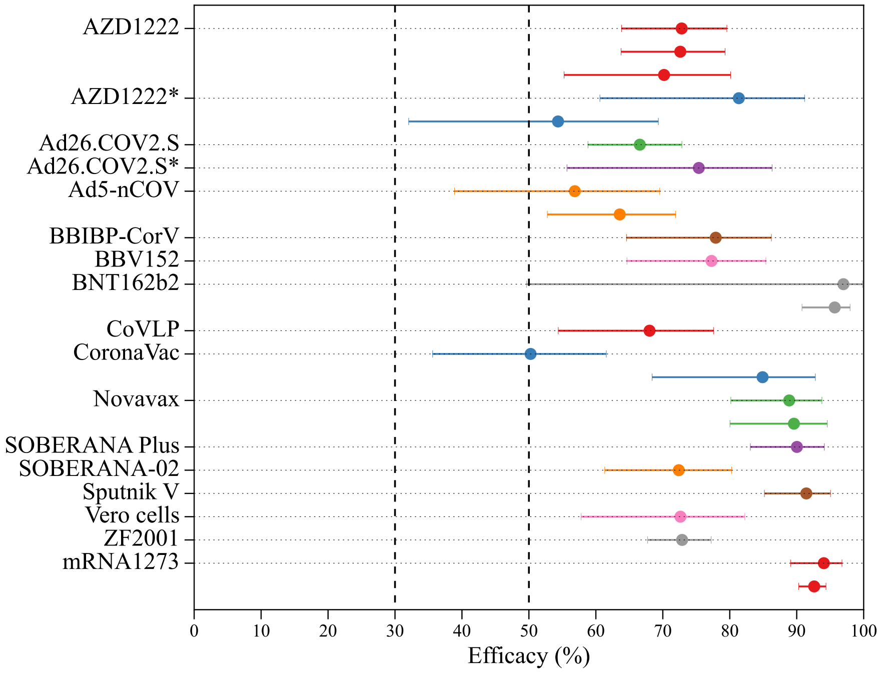

Fig. 15: Vaccine efficacy against SARS-CoV-2 induced symptomatic endpoint with 95% confidence interval. The vaccine marked with * are the booster-dose vaccine. The 0.5 adjustment has been applied.

efficacy of, while CoVLP reported vaccine efficacy of 75.8% with 95%CI, 47.6-88.8%. The average vaccine efficacy of all vaccines is 66.95% (95% CI, 59.52-73.03) against induced moderate endpoint due to SARS-CoV-2.

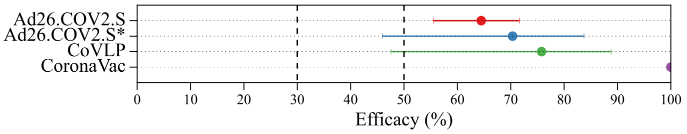

Fig. 16: Vaccine efficacy against SARS-CoV-2 induced moderate endpoint with 95% confidence interval. The vaccine marked with * is a booster dose vaccine.

Figure 18 is the same as the main text Figure 7 but with the 0.5 method.

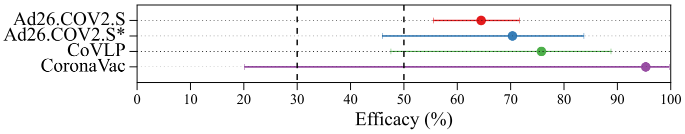

Fig. 17: Vaccine efficacy against SARS-CoV-2 induced moderate endpoint with 95% confidence interval. The vaccine marked with * is a booster dose vaccine. The 0.5 adjustment has been applied.

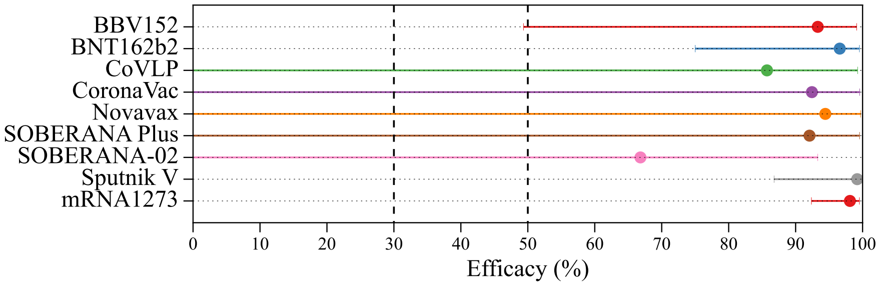

Fig. 18: Vaccine efficacy against SARS-CoV-2 induced severe endpoint with 95% confidence interval. The 0.5 adjustment has been applied.

We identified one data point in figure 19 of the BNT162b2 vaccine against documented SARS-CoV-2 infection. The BNT162b2 vaccine showed vaccine efficacy of 90.5% with 95% CI 88.1-92.4% [47].

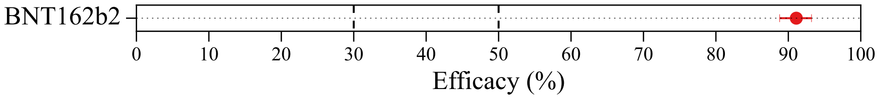

Fig. 19: Vaccine efficacy against SARS-CoV-2 induced documented endpoint infection with 95% confidence interval.

In Figure 20, one vaccine reported vaccine efficacy against SARS-CoV-2 hospitalization. In the phase III clinical trial, AZD1222 had a vaccine efficacy of 94.0% with 95%CI, 51.6-99.2% [7].

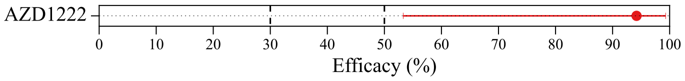

Fig. 20: Vaccine efficacy against SARS-CoV-2 induced hospitalization endpoint with 95% confidence interval.

The AZD1222 vaccines' phase 3 clinical trial also reported vaccine efficacy of 94.6% with 95%CI, 57.6-99.3% against ED visits (figure 21) and vaccine efficacy of 100% against the ICU (figure 22) due to SARS-CoV-2 [7]. Note that Falsey et al. estimated an alarming 95%CI, -1781.6-100% against ICU in the original study.

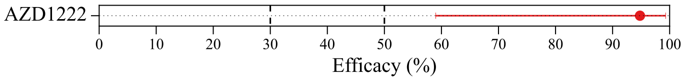

Fig. 21: Vaccine efficacy against SARS-CoV-2 induced ED endpoint visits with 95% confidence interval.

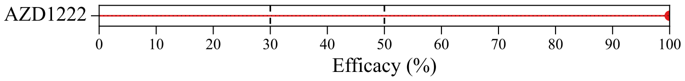

Fig. 22: Vaccine efficacy against SARS-CoV-2 induced ICU endpoint with 95% confidence interval.

We obtained four endpoints for vaccine efficacy against critical SARS-CoV-2 from 3 phase III clinical trials, as shown in figure 24. All of the vaccines demonstrated vaccine efficacy of greater than 70% and AZD1222 and booster dose vaccine Ad.COV2.S vaccine shows vaccine efficacy of 100%. Against the critical endpoint measure, we found that the average vaccine efficacy of all vaccines is 86.05% (95% CI, 77.62-91.31%).

We retrieved three endpoints of vaccine efficacy against death due to SARS-CoV-2 from two vaccines and a booster dose vaccine represented in Figure 26. Soberana plus, a booster dose vaccine reported 100% VE without CI with no deaths reported

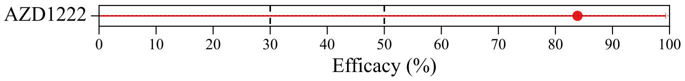

Fig. 23: Vaccine efficacy against SARS-CoV-2 induced ICU endpoint with 95% confidence interval. The 0.5 adjustment has been applied.

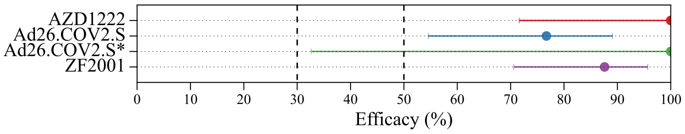

Fig. 24: Vaccine efficacy against SARS-CoV-2 induced critical endpoint with 95% confidence interval. The vaccine marked with * is a booster dose vaccine.

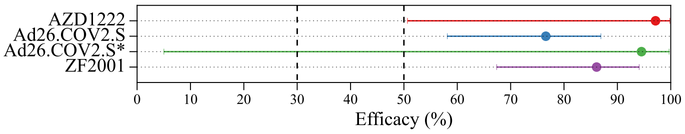

Fig. 25: Vaccine efficacy against SARS-CoV-2 induced critical endpoint with 95% confidence interval. The vaccine marked with * is a booster dose vaccine. The 0.5 adjustment has been applied.

in the vaccine group and 8 deaths in the placebo With 5 deaths in the vaccine group and 28 deaths in the placebo group across all endpoints, the overall vaccine efficacy of measure endpoint death is 82.01% (95% CI, 53.44-93.05%).

We also compared the vaccine efficacy categorized by platform, shown as forest plots in Figures 28 to 33. The overall vaccine efficacy for each category is $67.3(45.8, 80.2)\%$ for DNA, $69.6(64.5, 73.9)\%$ for Inactivated, $74.1(71.8, 76.3)\%$ for NRVV, $78.1(75.6, 80.3)\%$ for Protein subunit, $86.1(84.5, 87.5)\%$ for RNA, and $70.2(58.9, 78.4)\%$ for VLP respectively.

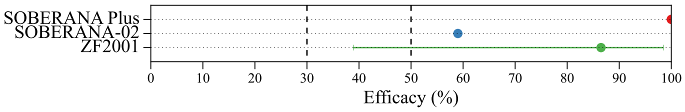

Fig. 26: Vaccine efficacy against SARS-CoV-2 induced death endpoint with 95% confidence interval.

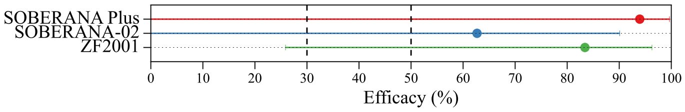

Fig. 27: Vaccine efficacy against SARS-CoV-2 induced death endpoint with 95% confidence interval. The 0.5 adjustment has been applied.

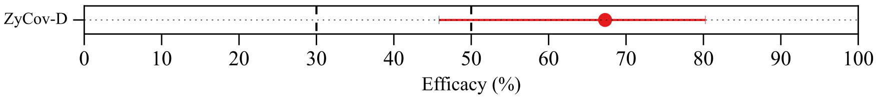

Fig. 28: Vaccine efficacy with 95% confidence interval of DNA platform.

9.1 Interim vaccine efficacy

15 vaccines did not publish their phase III clinical trial studies, out of the 38 vaccines that have been approved. To find out the interim vaccine efficacy of the approved vaccines, we searched the company website, Wikipedia pages, and media reports. Only three vaccine manufacturers provided interim vaccine efficacy data without confidence intervals. The Center for Genetic Engineering and Biotechnology in Cuba developed the CIGB-66 vaccine. The vaccine had a reported efficacy of 92.28% after enrolling approximately 48920 participants in both the vaccine and placebo groups [48]. According to preliminary data, Russia's CoviVac vaccine, which has two doses that can be given 14 days apart, has an interim vaccine efficacy of 80% [49]. EpivacCorona enrolled 2999 people in its phase III clinical trial, with 75% of them receiving the vaccine. Overall, the vaccine had an interim efficacy of 79% [50].

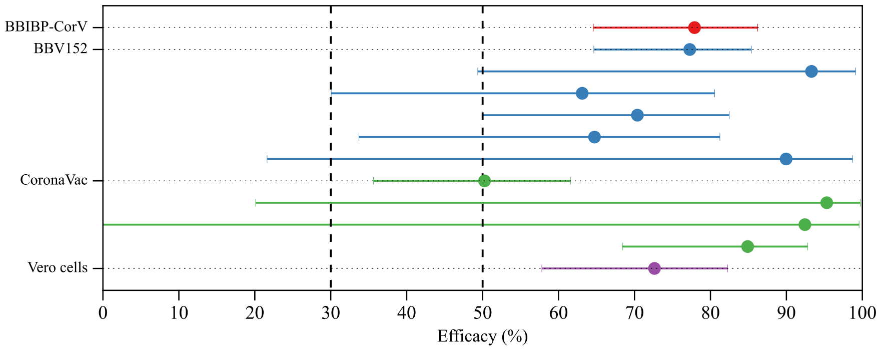

Fig. 29: Vaccine efficacy with 95% confidence interval of inactivated platform.

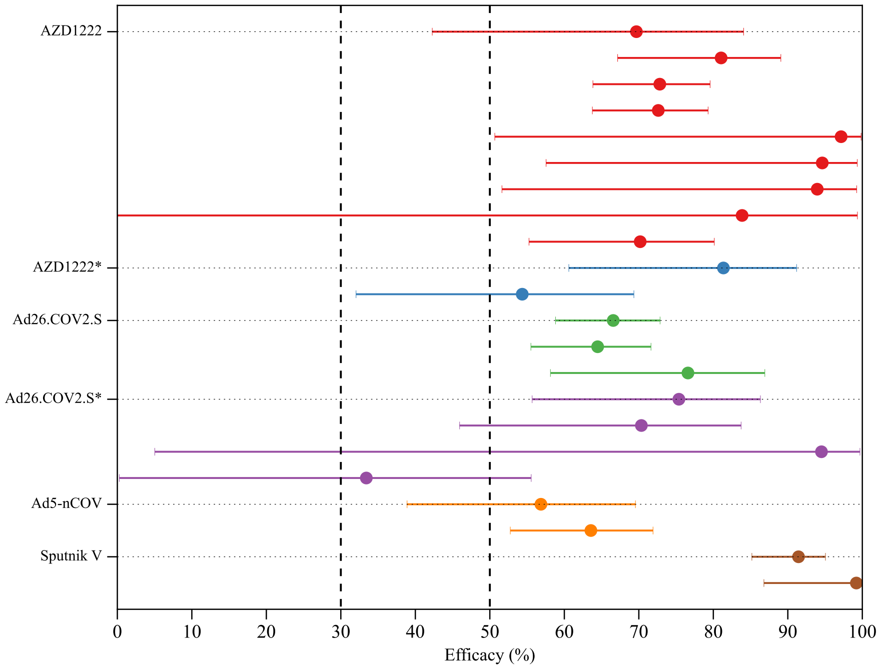

Fig. 30: Vaccine efficacy with 95% confidence interval of Non Replicating Viral Vector (NRVV) platform.

Out of the remaining fifteen vaccines, three vaccines have released interim vaccine efficacy through the press conference, media reports or on the company website. After reviewing all of the articles and their supplementary, we found that the author [21]

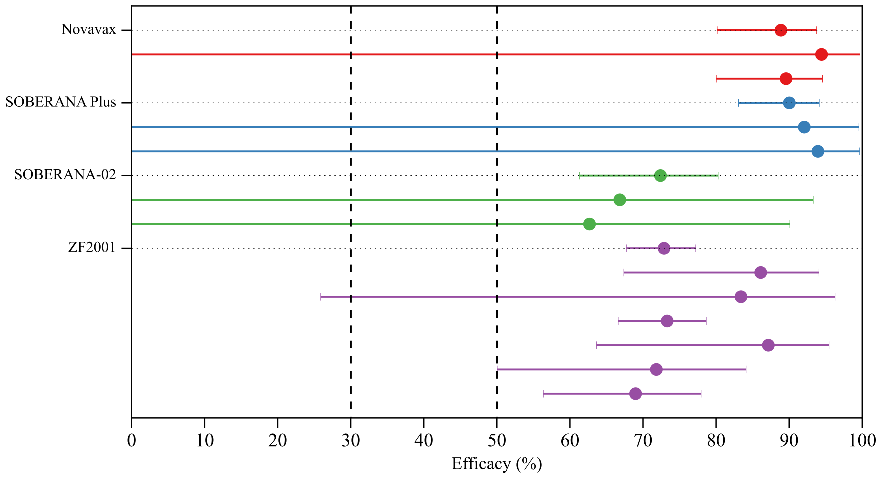

Fig. 31: Vaccine efficacy with 95% confidence interval of protein subunit platform.

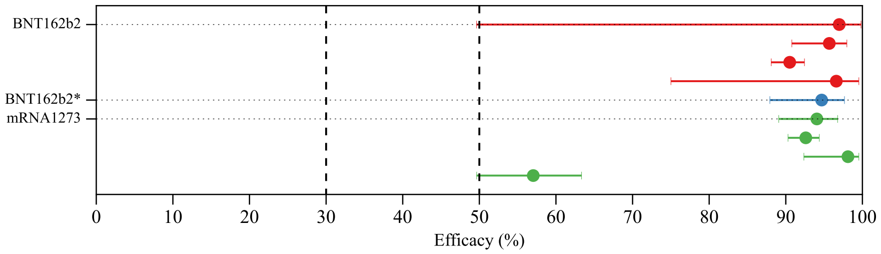

Fig. 32: Vaccine efficacy with 95% confidence interval of RNA platform.

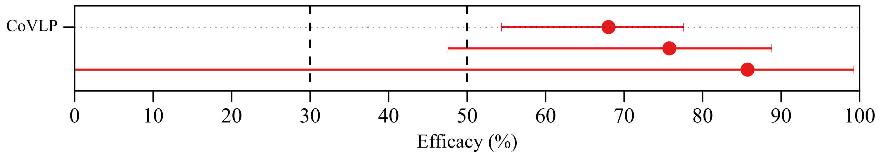

Fig. 33: Vaccine efficacy with 95% confidence interval of vlp platform.

did not specify the methods used to estimate vaccine efficacy in their article. The author [29] calculates the vaccine efficacy of Zy-CoV-D using the incidence rate ratio, but no method for calculating confidence intervals is mentioned. The authors, [6] and

[22] determine vaccine efficacy using the hazard ratio but do not indicate how they compute the confidence interval.

References

[1] González S, Olszevicki S, Salazar M, Calabria A, Regairaz L, Marín L, et al. Effectiveness of the first component of Gam-COVID-Vac (Sputnik V) on reduction of SARS-CoV-2 confirmed infections, hospitalisations and mortality in patients aged 60-79: a retrospective cohort study in Argentina. EClinicalMedicine. 2021;40:101126.

[2] Ella R, Reddy S, Blackwelder W, Potdar V, Yadav P, Sarangi V, et al. Efficacy, safety, and lot-to-lot immunogenicity of an inactivated SARS-CoV-2 vaccine (BBV152): interim results of a randomised, double-blind, controlled, phase 3 trial. The Lancet. 2021;398(10317):2173–2184.

[3] Dunkle LM, Kotloff KL, Gay CL, Áñez G, Adelglass JM, Barrat Hernández AQ, et al. Efficacy and Safety of NVX-CoV2373 in Adults in the United States and Mexico. New England Journal of Medicine. 2022;386(6):531–543.

[4] Sadoff J, Gray G, Vandebosch A, Cárdenas V, Shukarev G, Grinsztejn B, et al. Safety and efficacy of single-dose Ad26. COV2. S vaccine against Covid-19. New England Journal of Medicine. 2021;(23):2187–2201.

[5] Logunov DY, Dolzhikova IV, Shcheblyakov DV, Tukhvatulin AI, Zubkova OV, Dzharullaeva AS, et al. Safety and efficacy of an rAd26 and rAd5 vector-based

heterologous prime-boost COVID-19 vaccine: an interim analysis of a randomised controlled phase 3 trial in Russia. The Lancet. 2021;397(10275):671–681.

[6] Goldberg Y, Mandel M, Woodbridge Y, Fluss R, Novikov I, Yaari R, et al. Similarity of Protection Conferred by Previous SARS-CoV-2 Infection and by BNT162b2 Vaccine: A 3-Month Nationwide Experience From Israel. American Journal of Epidemiology. 2022;191(8):1420–1428.

[7] Falsey AR, Sobieszczyk ME, Hirsch I, Sproule S, Robb ML, Corey L, et al. Phase 3 safety and efficacy of AZD1222 (ChAdOx1 nCoV-19) Covid-19 vaccine. New England Journal of Medicine. 2021;385(25):2348–2360.

[8] Heath PT, Galiza EP, Baxter DN, Boffito M, Browne D, Burns F, et al. Safety and efficacy of NVX-CoV2373 Covid-19 vaccine. New England Journal of Medicine. 2021;385(13):1172–1183.

[9] Fu W, Li J, Scheet P. Covid-19 vaccine efficacy: accuracy, uncertainty and projection of cases. medRxiv. 2020;.

[10] Zou G. A modified poisson regression approach to prospective studies with binary data. American journal of epidemiology. 2004;159(7):702–706. Google Scholar citations: 5,865 (checked 2025-02-26); Highly cited. https://doi.org/10.1093/aje/kwh090.

[11] Pagano M, Gauvreau K, Mattie H. Principles of Biostatistics. 3rd ed. Belmont, CA: Chapman & Hall/CRC; 2022. Google Scholar citations for all editions: ¿5,600

(checked 2025-02-26); Highly cited. Available from: https://www.routledge.com/Principles-of-Biostatistics/Pagano-Gauvreau-Mattie/p/book/9780367345525.

[12] Palacios R, Batista AP, Albuquerque CSN, Patio EG, do Prado Santos J, Conde MTRP, et al. Efficacy and Safety of a COVID-19 Inactivated Vaccine in Healthcare Professionals in Brazil: The PROFISCOV Study. SSRN Electronic Journal. 2021 4;https://doi.org/10.2139/SSRN.3822780.

[13] Sterne JA, Sutton AJ, Ioannidis JP, Terrin N, Jones DR, Lau J, et al. Recommendations for examining and interpreting funnel plot asymmetry in meta-analyses of randomised controlled trials. Bmj. 2011;343.

[14] Frenck Jr RW, Klein NP, Kitchin N, Gurtman A, Absalon J, Lockhart S, et al. Safety, immunogenicity, and efficacy of the BNT162b2 Covid-19 vaccine in adolescents. New England Journal of Medicine. 2021;385(3):239–250.

[15] Voysey M, Clemens SAC, Madhi SA, Weckx LY, Folegatti PM, Aley PK, et al. Safety and efficacy of the ChAdOx1 nCoV-19 vaccine (AZD1222) against SARS-CoV-2: an interim analysis of four randomised controlled trials in Brazil, South Africa, and the UK. The Lancet. 2021;397(10269):99–111.

[16] Hardt K, Vandebosch A, Sadoff J, Le Gars M, Truyers C, Lowson D, et al. Efficacy, safety, and immunogenicity of a booster regimen of Ad26. COV2. S vaccine against COVID-19 (ENSEMBLE2): results of a randomised, double-blind, placebo-controlled, phase 3 trial. The Lancet Infectious Diseases. 2022;22(12):1703–1715.

[17] Halperin SA, Ye L, MacKinnon-Cameron D, Smith B, Cahn PE, Ruiz-Palacios GM, et al. Final efficacy analysis, interim safety analysis, and immunogenicity of a single dose of recombinant novel coronavirus vaccine (adenovirus type 5 vector) in adults 18 years and older: an international, multicentre, randomised, double-blinded, placebo-controlled phase 3 trial. The Lancet. 2022;399(10321):237–248.

[18] Al Kaabi N, Zhang Y, Xia S, Yang Y, Al Qahtani MM, Abdulrazzaq N, et al. Effect of 2 Inactivated SARS-CoV-2 Vaccines on Symptomatic COVID-19 Infection in Adults: A Randomized Clinical Trial. JAMA. 2021;(1):35–45.

[19] Polack FP, Thomas SJ, Kitchin N, Absalon J, Gurtman A, Lockhart S, et al. Safety and efficacy of the BNT162b2 mRNA Covid-19 vaccine. New England Journal of Medicine. 2020;383(27):2603–2615.

[20] Hager KJ, Pérez Marc G, Gobeil P, Diaz RS, Heizer G, Llapur C, et al. Efficacy and Safety of a Recombinant Plant-Based Adjuvanted Covid-19 Vaccine. New England Journal of Medicine. 2022;386(22):2084–2096.

[21] Tanriover MD, Doğanay HL, Akova M, Güner HR, Azap A, Akhan S, et al. Efficacy and safety of an inactivated whole-virion SARS-CoV-2 vaccine (CoronaVac): interim results of a double-blind, randomised, placebo-controlled, phase 3 trial in Turkey. The Lancet. 2021;398(10296):213–222.

[22] Toledo-Romani ME, Garcia-Carmenate M, Silva CV, Baldoquin-Rodriguez W, Perez MM, Gonzalez MCR, et al. Efficacy and Safety of SOBERANA 02, a COVID-19 conjugate vaccine in heterologous three doses combination. medRxiv.

2021;.

[23] Dai L, Gao L, Tao L, Hadinegoro SR, Erkin M, Ying Z, et al. Efficacy and Safety of the RBD-Dimer–Based Covid-19 Vaccine ZF2001 in Adults. New England Journal of Medicine. 2022;386(22):2097–2111.

[24] El Sahly HM, Baden LR, Essink B, Doblecki-Lewis S, Martin JM, Anderson EJ, et al. Efficacy of the mRNA-1273 SARS-CoV-2 vaccine at completion of blinded phase. New England Journal of Medicine. 2021;385(19):1774–1785.

[25] Baden LR, El Sahly HM, Essink B, Kotloff K, Frey S, Novak R, et al. Efficacy and safety of the mRNA-1273 SARS-CoV-2 vaccine. New England Journal of Medicine. 2021;384(5):403–416.

[26] Emary KR, Golubchik T, Aley PK, Ariani CV, Angus B, Bibi S, et al. Efficacy of ChAdOx1 nCoV-19 (AZD1222) vaccine against SARS-CoV-2 variant of concern 202012/01 (B. 1.1. 7): an exploratory analysis of a randomised controlled trial. The Lancet. 2021;397(10282):1351–1362.

[27] Moreira Jr ED, Kitchin N, Xu X, Dychter SS, Lockhart S, Gurtman A, et al. Safety and efficacy of a third dose of BNT162b2 COVID-19 vaccine. New England Journal of Medicine. 2022;386(20):1910–1921.

[28] Kremsner PG, Guerrero RAA, Arana E, Aroca Martinez GJ, Bonten MJ, Chandler R, et al. Efficacy and Safety of the CVnCoV SARS-CoV-2 mRNA vaccine candidate: results from Herald, a phase 2b/3, randomised, observer-blinded,

placebo-controlled clinical trial in ten countries in Europe and Latin America. New England Journal of Medicine. 2021;22(3):329–340.

[29] Khobragade A, Bhate S, Ramaiah V, Deshpande S, Giri K, Phophle H, et al. Efficacy, safety, and immunogenicity of the DNA SARS-CoV-2 vaccine (ZyCoV-D): the interim efficacy results of a phase 3, randomised, double-blind, placebo-controlled study in India. The Lancet. 2022;399(10332):1313–1321.

[30] Shrestha Y, Venkataraman R, Moktan JB, Chitti R, Yadav SK. COVID-19 Vaccine Authorized in India-A Mini Review. SSRN Electronic Journal. 2021 4;https://doi.org/10.2139/SSRN.3836545.

[31] Nicole E Basta .: McGill University COVID19 Vaccine Tracker. Last visited on 2022-06-10. Available from: https://covid19.trackvaccines.org/our-team/.

[32] FDA.: FDA In Brief: FDA Authorizes Longer Time for Refrigerator Storage of Thawed Pfizer-BioNTech COVID-19 Vaccine Prior to Dilution, Making Vaccine More Widely Available. Visited on 2021-07-26. Available from: https://www.fda.gov/news-events/press-announcements/fda-brief-fda-authorizes-longer-time-refrigerator-storage-thawed-pfizer-biontech-covid-19-vaccine/.

[33] Griffiths E, Tanner J, Knox N, Hsiao W, Van Domselaar G. CanCOGeN Interim Recommendations for Naming, Identifying, and Reporting SARS-CoV-2 Variants of Concern. NCCID. 2021;1.

[34] Lucey DR.: COVID Mega-variant and eight criteria for a template to assess all variants. Visited on 2021-06-29. Available from: https://sciencespeaksblog.org/2021/02/02/covid-mega-variant-and-eight-criteria-for-a-template-to-assess-all-variants///.

[35] Liu T, Chen Z, Chen W, Chen X, Asanjan MH, Yang Z, et al. A benchmarking study of SARS-CoV-2 whole-genome sequencing protocols using COVID-19 patient samples. bioRxiv. 2020;.

[36] WHO.: Classification of Omicron (B.1.1.529): SARS-CoV-2 Variant of Concern. Last visited on 2021-11-29. Available from: https://www.who.int/news/item/26-11-2021-classification-of-omicron-(b.1.1.529)-sars-cov-2-variant-of-concern.

[37] Davies NG, Abbott S, Barnard RC, Jarvis CI, Kucharski AJ, Munday JD, et al. Estimated transmissibility and impact of SARS-CoV-2 lineage B. 1.1. 7 in England. Science. 2021;372(6538).

[38] Challen R, Brooks-Pollock E, Read JM, Dyson L, Tsaneva-Atanasova K, Danon L. Risk of mortality in patients infected with SARS-CoV-2 variant of concern 202012/1: matched cohort study. bmj. 2021;372.

[39] WHO. COVID-19 weekly epidemiological update, edition 45, 22 June 2021. WHO news letter. 2021;.

[40] TWR CAP, Davies NG, Kucharski AJ, working group C, Edmunds WJ, Eggo RM, et al. Estimates of severity and transmissibility of novel South Africa SARS-CoV-2 variant501Y. V2 Centre for Mathematical Modelling of Infectious Diseases. 2021;.

[41] Coutinho RM, Marquitti FMD, Ferreira LS, Borges ME, da Silva RLP, Canton O, et al. Model-based estimation of transmissibility and reinfection of SARS-CoV-2 P. 1 variant. medRxiv. 2021;.

[42] Faria NR, Mellan TA, Whittaker C, Claro IM, Candido DdS, Mishra S, et al. Genomics and epidemiology of the P. 1 SARS-CoV-2 lineage in Manaus, Brazil. Science. 2021;372(6544):815–821.

[43] Richer S.: Covid-19: What should we do about B.1.617.2? A classic case of decision making under uncertainty. Visited on 2021-05-17. Available from: https://blogs.bmj.com/bmj/2021/05/17/covid-19-what-should-we-do-about-b-1-617-2-a-classic-case-of-decision-making-under-uncertainty/.

[44] for Disease Control C, Prevention, et al. SARS-CoV-2 variant classifications and definitions. Retrieved March. 2021;16:2020.

[45] WHO.: Tracking SARS-CoV-2 variants. Visited on 2022-06-16. Available from: https://www.who.int/activities/tracking-SARS-CoV-2-variants/.

[46] Deng X, Garcia-Knight MA, Khalid MM, Servellita V, Wang C, Morris MK, et al. Transmission, infectivity, and antibody neutralization of an emerging SARS-CoV-2 variant in California carrying a L452R spike protein mutation. medRxiv. 2021;.

[47] Thomas SJ, Moreira Jr ED, Kitchin N, Absalon J, Gurtman A, Lockhart S, et al. Safety and efficacy of the BNT162b2 mRNA Covid-19 vaccine through 6 months. New England Journal of Medicine. 2021;385(19):1761–1773.

[48] Reuters.: Cuba says Abdala vaccine 92.28% effective against coronavirus. Visited on 2022-04-28. Available from: https://www.reuters.com/business/healthcare-pharmaceuticals/cuba-says-abdala-vaccine-9228-effective-against-coronavirus-2021-06-21/.

[49] Reuters.: Russia's CoviVac more than 80% effective against COVID-19. Visited on 2022-04-17. Available from: https://www.reuters.com/business/healthcare-pharmaceuticals/russias-covivac-more-than-80-effective-against-covid-19-ifax-2021-06-02/.

[50] TASS.: EpivacCorona vaccines immunological efficacy proves to be 79% . Visited on 2022-04-17. Available from: https://tass.com/society/1322797/.
